# Black soybean seed coat polyphenol ameliorates the abnormal feeding pattern induced by high-fat diet consumption

**DOI:** 10.3389/fnut.2022.1006132

**Published:** 2022-10-10

**Authors:** Ken-yu Hironao, Hitoshi Ashida, Yoko Yamashita

**Affiliations:** Department of Agrobioscience, Graduate School of Agricultural Science, Kobe University, Kobe, Japan

**Keywords:** obesity, feeding rhythm, cyanidin 3-*O*-glucoside, hypothalamus, microglia, inflammation, high-fat diet, polyphenol

## Abstract

High-fat diet (HFD) consumption induces chronic inflammation and microglial accumulation in the mediobasal hypothalamus (MBH), the central regulator of feeding behavior and peripheral metabolism. As a result, the diurnal feeding rhythm is disrupted, leading to the development of obesity. Diet-induced obesity (DIO) can be prevented by restoring the normal feeding pattern. Therefore, functional foods and drugs that ameliorate hypothalamic inflammation and restore the normal feeding pattern may prevent or ameliorate DIO. Numerous functional foods and food-derived compounds with anti-obesity effects have been identified; however, few studies have been performed that assessed their potential to prevent the HFD-induced hypothalamic inflammation and disruption of feeding rhythm. In the present study, we found that polyphenols derived from black soybean seed coat (BE) significantly ameliorated the accumulation of activated microglia and pro-inflammatory cytokine expression in the arcuate nucleus of the hypothalamus of HFD-fed mice, and restored their feeding pattern to one comparable to that of standard diet-fed mice, thereby ameliorating DIO. Furthermore, cyanidin 3-*O*-glucoside—the principal anthocyanin in BE—was found to be a strong candidate mediator of these effects. This is the first study to show that BE has the potential to provide a variety of beneficial effects on health, which involve amelioration of the HFD-induced hypothalamic inflammation and abnormal feeding pattern. The results of this study provide new evidence for the anti-obesity effects of black soybean polyphenols.

## Introduction

Diet-induced obesity (DIO) is the result of both the ingestion of inappropriate foods and eating at inappropriate times (1). Rodents normally feed during the dark/active period, rather than in the light/inactive period. Rodents fed an HFD *ad libitum* show an abnormal feeding pattern, involving hyperphagia during the light period, and develop obesity, diabetes, and metabolic syndrome (2). In contrast, HFD-feeding that is restricted to the dark period does not result in diet-induced obesity (DIO) (3), and the feeding of a standard diet during the light period does causes obesity (4, 5). In addition, repeated food consumption at the inactive period increases the risk of obesity and various other metabolic diseases in humans (6–8). These lines of evidence suggest that improperly timed meals, rather than the contents of the meals, are the principal cause of DIO. Therefore, DIO may be best prevented by maintaining appropriate meal timing. To further justify this approach, it is essential to understand how HFD consumption affects the *ad libitum* circadian feeding pattern.

Previous studies have shown that the abnormal feeding rhythm associated with HFD consumption is caused by hypothalamic inflammation (9, 10). Hypothalamic microglia, which play a macrophage-like role in the brain, are activated by long-chain saturated fatty acids, such as palmitic acid, a component of HFDs (9, 10). Microglial activation in the hypothalamic arcuate nucleus (ARC), the center for the control of feeding behavior, interferes with the function of neurons involved in appetite regulation, which disrupts the feeding rhythm (9, 11). This sequence of events occurs within just a few days of starting the consumption of an HFD (11), and HFD consumption for several months causes chronic inflammation, characterized by activation of the NF-κB pathway (12, 13). Chronic inflammation in the ARC reduces neuronal sensitivity to nutrients and hormones (9, 14, 15), leading to the dysregulation of feeding behavior and impairs peripheral metabolism (16, 17). The prevention of neuroinflammation mediated by activated microglia has been shown to improve the altered feeding pattern and suppress obesity (9, 11, 13, 15, 18). Thus, hypothalamic inflammation plays a key role in the pathogenesis of HFD-induced obesity, and its suppression might have anti-obesity effects.

Both anti-obesity drugs and functional food materials have been developed for the treatment of obesity, in addition to exercise and diet therapy. These functional foods include dietary antioxidants, such as polyphenols, alkaloids, isothiocyanates, vitamins, and carotenoids, which have anti-obesity effects (19, 20). Of the polyphenols, epigallocatechin gallate (21) and kaempferol (22) have been shown to reduce the hypothalamic microglial inflammation induced by HFD consumption in mice. Cyanidin 3-*O*-glucoside (C3G) and its metabolite, protocatechuic acid, suppress lipopolysaccharide-induced microglial inflammation in the VB-2 microglial cell line (23, 24). In addition, myricetin has been reported to inhibit microglial activation in hypoxic VB-2 cells (25). These results indicate that polyphenols and polyphenol-rich food materials might have both anti-obesity and anti-inflammatory effects. However, no studies have investigated the effects of functional foods on the combination of hypothalamic inflammation, abnormal feeding rhythm, and obesity.

We have obtained a polyphenol-rich extract from the seed coat of black soybean (*Glycine max L*) (BE), which is rich in (+)-catechin, (–)-epicatechin, and their polymeric procyanidins (PCAs), as well as anthocyanins, principally C3G. Previous studies have shown that BE has various beneficial effects, including anti-hyperglycemic (26), antioxidant (27), and anti-obesity (28) effects; an improvement in vascular function (29, 30); and the prevention of non-alcoholic fatty liver disease and non-alcoholic steatohepatitis (31). We have previously shown that BE increases the expression of uncoupling proteins, which are responsible for heat production in brown/beige adipose tissue, and has an anti-obesity effect (28). However, the effects of this preparation on the abnormal feeding patterns and hypothalamic inflammation of models of obesity have not been evaluated. The hypothalamus is a central regulator not only of feeding behavior but also of the metabolism of peripheral adipose tissue and skeletal muscle (32). Therefore, food ingredients that reduce obesity and hyperglycemia might achieve these effects through a suppression of hypothalamic inflammation. In the present study, we aimed to determine whether BE would prevent the effects of HFD consumption on the feeding pattern and hypothalamic inflammation of rodents. In this way, we aimed to identify novel potential mechanisms for the anti-obesity effects of BE and provide new evidence for the effects of polyphenols on the central nervous system (CNS).

## Materials and methods

### Chemicals and reagents

BE was obtained from Fujicco Co., Ltd. (Kobe, Japan). The polyphenol composition of this BE was 9.2% cyanidin 3-*O*-glucoside (C3G), 6.2 EC, and 39.7% PCAs, including 6.1 dimers, 3.4 trimers, 0.5% tetramers, and higher degree of polymerized PCAs, which are more polymerized than tetramers; determined using high-performance liquid-chromatography (HPLC) and expressed as mass/mass ratio as previously described (28, 33). Silica gel (Chromatorex; #PSQ100B) was purchased from Fuji Silysia Chemical Ltd. (Aichi, Japan). Tissue-Tek^®^ Paraffin WaxII60 was purchased from Sakura Finetek Japan Co., Ltd. (Tokyo, Japan). Glass slides (Crest Coat; SCRE-01) were purchased from Matsunami Glass Ind., Ltd. (Osaka, Japan). Methanol (HPLC grade) and fatty acid-free bovine serum albumin (BSA) (#013-15143) were purchased from Fujifilm Wako Pure Chemical Co., Ltd. (Osaka, Japan). Fetal bovine serum (FBS) was purchased from BioWest S.A.S. (Nuaillé, France). Domitor^®^ (1.0 mg/ml medetomidine hydrochloride) was purchased from Nippon Zenyaku Kogyo Co., Ltd. (Fukushima, Japan). Pentobarbital sodium salt (#P0776) was purchased from Tokyo Chemical Industry Co., Ltd. (Tokyo, Japan). Blocking One (#03953-95) and Blocking One-P (#05999-84) were purchased from Nacalai Tesque Inc. (Kyoto, Japan). Primary antibodies for western blotting; anti-heat-shock protein 70 (Hsp70) rabbit polyclonal antibody (#4872), anti-β-actin rabbit polyclonal antibody (#4967), anti-nuclear factor-kappa B (NF-κB) p65 rabbit polyclonal antibody (#3034), anti-phospho-NF-κB p65 (Ser536) (93H1) rabbit monoclonal antibody (#3033), anti-inhibitor of nuclear factor-kappa B alpha (IκBα) rabbit polyclonal antibody (#9242), anti-phospho-IκBα (Ser32/36) (5A5) mouse monoclonal antibody (#9246), and anti-glial fibrillary acidic protein (GFAP) (5GA) mouse monoclonal antibody (#3670), and secondary antibodies for western blotting; goat anti-rabbit IgG horseradish peroxidase-linked antibody (#7074), and horse anti-mouse IgG horseradish peroxidase-linked antibody (#7076) were purchased from Cell Signaling Technology Co., Ltd. (Danvers, MA, USA). All the other reagents used were of the highest grade available from commercial sources.

### Separation of C3G, flavan 3-ols monomers, and the polymer fraction of BE

First, to extract the C3G and flavan 3-ols from BE, 10 g of BE powder was suspended in 100 ml of 0.1% (w/v) HCl and subjected to extraction using double volume of ethyl acetate. This process was repeated four times. The aqueous layer containing C3G (C3G-rich fraction) and the ethyl acetate fraction containing the flavan 3-ols were obtained and dried *in vacuo*. The C3G-rich fraction was subjected to HPLC separation under the conditions described in the Section Separation and purification of C3G using HPLC, to obtain 98% (w/w) pure C3G, while the ethyl acetate fraction was subjected to a silica gel column chromatography (5 × 75 cm, 100 μm mesh silica gel, charged with chloroform/methanol =4:1) to obtain a fraction containing monomeric flavan 3-ols, catechin, and epicatechin; and another containing the dimeric and higher-degree of polymerized PCAs. Elution was performed with mobile phase consisting of chloroform/methanol =4:1, and eluate was collected 20 ml each in test tubes. Each fraction was checked by a thin layer chromatography using a silica gel 60 F254 (#105549; Merck, Darmstadt, Germany) and chloroform/methanol =4:1, alongside flavan 3-ol standards. To detect the compounds in each fraction, the plate was exposed to UV light at a wavelength of 254 nm. The eluates containing catechin and epicatechin were combined, and the solvent was evaporated by a rotary evaporator. Obtained fraction was referred to as the Epicatechin (EC)-rich fraction. The eluates containing procyanidin B2, procyanidin C1, and cinnamtannin A2 were also evaporated separately, and this fraction was referred to as the PCA-rich fraction. The amount of each flavan 3-ol in the fraction was measured using a HPLC method (33): the EC fraction contained 64.98% (–)-epicatechin and 2.57% (+)-catechin; while the PCA fraction contained 39.13% procyanidin B2, 20.74% procyanidin C1, and 8.97% cinnamtannin A2. The HPLC chromatogram of EC-rich, PCA-rich and C3G-rich fractions were described in Supplementary Figures S6, S7.

### Separation and purification of C3G using HPLC

Preparative HPLC was performed using a Shimadzu LabSolutions system (Shimadzu, Kyoto, Japan), an SPD-M20A photodiode array detector, a CTO-20A column oven, a CBM-20A communications bus module, and an LC-20AD binary pump. HPLC separation was performed with a gradient system using 0.1% (v/v) trifluoroacetic acid in water as mobile phase A and 0.1% (v/v), trifluoroacetic acid in methanol as mobile phase B, a Tsk-gel ODS-80Ts (20 mm × 250 mm, 5 μm; #0018409; Tosoh Co., Ltd., Tokyo, Japan), and a flow rate of 8.0 ml/min. The injection volume was 500 μL and the temperature of the column oven was maintained at 40°C. C3G was separated using a linear gradient, commencing with 33% B over 0–22.5 min; then followed by 90% B over 22.5–35 min, and 33% B over 35–50 min, with elution between 16 and 22 min. The eluate was collected and evaporated to obtain purified-C3G. The gradient from 35 min onwards was used to re-equilibrate the system between samples. The absorbance of C3G was monitored at 280 nm and 513 nm using a UV detector. The HPLC chromatogram of purified-C3G was described in Supplementary Figure S7.

### Animal experiments

The animal experiments were approved by the Institutional Animal Care and Use Committee (approval number: 2020-10-13) and performed in accordance with the Guidelines for Animal Experiments of Kobe University. Male, 5-week-old C57BL/6J mice were purchased from Japan SLC, Inc. (Shizuoka, Japan), and were kept in a temperature- and humidity-controlled room (temperature: 23 ± 2°C, humidity: 50 ± 10%) under a 12:12–h light/dark cycle (lights on at 08:00). The mouse cages used were SEALSAFE^®^ GM500 (Tecniplast Co. Ltd., West Chester, PA, USA) with 501 cm^2^ floor area. Each mouse was housed in an individual cage. The mice were acclimatized to their environment for 1 week, with free access to standard diet [SD, containing 3.85 kcal/g, 10.0% kcal from fat (4.4 lard and 5.6% soybean oil), 20.0% kcal from protein, and 70.0% kcal from carbohydrates; #D12450J; Research Diets, Inc., New Brunswick, NJ, USA] and tap water. Their body weight and food intake were recorded periodically, at 08:00 (when the lights were turned on) or at 20:00 (when the lights were turned off), during the feeding period. The mice were used in the following two experiments and were fasted for 1 h before the sacrifice.

***Experiment 1:*** Ninety-six mice were allocated to three groups of thirty-two and were fed an SD (#D12450J; Research Diets, Inc., New Brunswick, NJ, USA), a high-fat diet [HFD, containing 5.24 kcal/g, 60.0% kcal from fat (54.4% lard, 5.6% soybean oil), 20.0% kcal from protein, and 10.0% kcal from carbohydrates; #D12492; Research Diets, Inc.], or the HFD supplemented with 2.0% (w/w) BE. Pellet-type SD and HFD were pulverized and gave animals. BE was mixed with powdered HFD as outer percentage. Each group was further divided into four sub-groups of eight each for 3-day, 1-, 2-, and 4-week groups. All mice were housed individually, and mice in the 4-week group were used for measurement of food intake. Five mice of each group were euthanized by exsanguination *via* cardiac puncture, and remaining three mice by systemic perfusion with 4% (w/v) paraformaldehyde (PFA) in phosphate-buffered saline (PBS) for immunohistochemical experiments, under anesthesia through an intraperitoneal injection of a mixture of sodium pentobarbital (65 mg/kg) as an anesthetic and medetomidine hydrochloride (0.3 mg/kg) as an analgesic. To obtain the hypothalamus, the collected brain was cut out with a range of bregma −1.00 to −2.50 using a rodent brain matrix (#RBM-2000C; Applied Scientific Instrumentation Inc., Eugene, OR, USA) on ice, under a binocular stereomicroscope. For immunohistochemistry, the cut-out coronal brain section was pre-fixed with 4% PFA-PBS; for total RNA and protein isolation, the hypothalamic block was obtained from the coronal section with the range of lateral −1.00 to 1.00 and interaural −0.50 to 2.00 using a scalpel. The hypothalamic block was stabilized in RNAlater^®^ (#R0901; Sigma-Aldrich, St Louis, MO, USA) and stored at 4°C, and total RNA and proteins were isolated within 1 week. The liver and adipose tissues were collected, weighed, and then immediately placed at −80°C.

***Experiment 2:*** Alternatively, thirty mice were allocated to five groups of six each, which were fed SD, HFD, and HFD containing 0.5% EC, 0.5% PCA, and 0.5% C3G for 4 weeks. Each component was mixed with the powdered diet as outer percentage and gave animals. At the end of the study, the mice were systemically perfused with 4% PFA in PBS under anesthesia as the same methods as ***Experiment 1***, and the brain was collected.

### Measurement of food intake

As for the feeding of diets, powdered diets (5.0 g/day/mice) were placed into a powder feeder (#MF-3S; Shin Factory Co., Ltd., Fukuoka, Japan) and replaced to fresh ones every 2 or 3 days. The amount of remained diets was weighed and calculated the food intake at the timing of each replacement. On days 0, 1, 3, 7, 14, 21, and 28 of the feedings, the food intake was measured during the 12 h of the light (inactive) and dark (active) periods.

### Isolation of RNA and RT-qPCR

RNA was isolated from hypothalamic blocks stored in RNA*later* using TRIzol™ Reagent (#15596018; Invitrogen, Carlsbad, CA, USA), following the manufacturer's instructions. The quality and concentration of the RNA obtained were measured by spectrophotometry using a NanoDrop™ ND-1,000 spectrophotometer (Thermo Fisher Scientific Co., Ltd., Waltham, MA, USA.). The RNA samples were purified by digesting the residual DNA using DNase I recombinant (#4716728001; Roche, Basel, Switzerland) following the manufacturer's instructions. The DNase-treated RNA was reverse transcribed to cDNA using ReverTra Ace^®^ (#TRT-101; Toyobo Co., Ltd.). cDNA was then subjected to RT-qPCR amplification using TB Green^®^ Premix Ex Taq™ II (#RR820; Takara Bio, Kusatsu, Japan). The primer sequences used are listed in Supplementary Table S1. Real-time PCR reactions were performed using a TaKaRa PCR Thermal Cycler Dice^®^ Real Time System II (#TP900; Takara Bio). Relative gene expression was calculated using the comparative CT method (34), using *Gapdh* as the reference gene. The results are expressed as fold-differences from the expression level of mice in the SD group.

### Protein isolation from hypothalamic blocks

Hypothalamic protein was isolated from hypothalamic blocks stored in RNA*later*^®^ using TRIzol™ Reagent, following the manufacturer's instructions. RNA and protein were isolated from the same mouse hypothalamic blocks. Briefly, 2-propanol was added to the phenol-containing layer to precipitate the protein, and the protein pellet was thoroughly washed with 95% ethanol containing 0.3 M guanidine hydrochloride. The obtained protein pellet was homogenized with RIPA buffer [50 mM Tris, pH8.0, containing 150 mM sodium chloride, 1% (v/v) Non-idet P-40 (NP-40), 0.5% (w/v) deoxycholic acid, 0.1% (w/v) sodium dodecyl sulfate and 0.5 mM dithiothreitol] using a Misonix™ Microson™ XL-2,000 ultrasonic homogenizer (Qsonica Co., Newtown, CT, USA). The homogenates were incubated on ice for 60 min, with occasional mixing, and then centrifuged at 12,000 × g for 20 min at 4°C. The supernatants were used as the protein lysates.

### Western blotting analysis

The protein concentrations of the obtained protein lysates were quantified using Lowry's method (35). Following this, the lysates were subjected to sodium dodecyl sulfate-polyacrylamide gel electrophoresis using 10% gels for the detection of Hsp70, β-actin, NF-κB p65, p-NF-κB p65 and GFAP; and 15% gels for the detection of IκBα and p-IκBα. The separated proteins were transferred onto Immobilon^®^-P polyvinylidene difluoride membranes (#IPVH00010; Merck Millipore Ltd., Billerica, MA, USA), which were incubated with blocking solutions [Blocking One for unphosphorylated proteins and Blocking One-P for phosphoproteins), then treated with primary antibodies (1:5,000) in Can Get Signal^®^ solution 1 (#NKB-101; Toyobo Co., Ltd., Osaka, Japan)] overnight at 4°C, and with the corresponding horseradish peroxidase-conjugated secondary antibody (1:50,000) in Can Get Signal^®^ solution 2 for 1 h at room temperature. Protein bands were visualized using ImmunoStar^®^ LD (#290-69904; Fujifilm Wako Pure Chemical, Osaka, Japan) and detected using LuminoGraphI (#WSE-6100; ATTO, Tokyo, Japan). Densitometric quantification of specific band was performed using ImageJ software (NIH, Bethesda, MD, USA).

### Immunohistochemistry

The anesthetized mice were perfused with 4% PFA in PBS for 1 h, and then their brains were collected and post-fixed in 4% PFA in PBS for 48 h at room temperature, before being processed for embedding in paraffin. Hypothalamic coronal sections (10-μm-thick) were prepared using a microtome (#PR-50; Yamato Kohki Industrial Co., Ltd., Asaka, Japan), deparaffinized, and rehydrated using standard techniques. Antigen retrieval was performed using 10 mM sodium citrate buffer (pH 6.0) at 95°C for 30 min. The sections were blocked with 10% (v/v) heat-inactivated FBS, 5% (w/v) BSA, and 0.1% (w/v) sodium azide in PBS containing 0.1% (v/v) Triton X-100 for 1 h at room temperature, and then incubated with primary antibody in PBS containing 0.05% (v/v) Tween-20 and 3% (w/v) BSA overnight at 4°C. Subsequently, the sections were washed three times with PBS containing 0.05% (v/v) Tween-20 and incubated with secondary antibody in the same solution as the primary antibody for 3 h at 4°C. The nuclei were counterstained with 10 μg/ml 4',6-diamidino-2-phenylindole. For the detection of Iba1 in the hypothalamic arcuate nucleus, a rabbit monoclonal [HL22] primary antibody against Iba1 (1:200; #GTX100042; GeneTex, Irvine, CA, USA) and an Alexa Fluor™ 488-conjugated goat anti-rabbit IgG (1:1,000; #A-11008; Invitrogen) were used. For the detection of GFAP, a mouse monoclonal primary antibody (GA5) against GFAP (1:1000; #3670; Cell Signaling Technology) and an Alexa Fluor™ 594-conjugated goat anti-mouse IgG (1:1,000; #A-11005; Invitrogen) were used. Images were acquired using a fluorescence microscope (#FSX100; Olympus, Tokyo, Japan). The numbers of cells that were immunoreactive for Iba1 on matched sections within prespecified regions of interest in the ARC (bregma −1.40 to −1.70 mm) of each mouse were determined using ImageJ software. To count the number of immunoreactive cells, the threshold was defined as the intensity at which the cells were clearly immunoreactive to Iba1 on visual inspection, and then they were counted manually and the mean values for three sections per mouse were calculated. The microglial cell (the cell type that is immunoreactive to Iba1) size was measured using a thresholding protocol (ImageJ), which was followed by densitometric quantification, performed according to the previously published method (36).

### Statistical analysis

Statistical analysis was performed using JMP statistical software, version 11.2.0. (SAS Institute, Cary, NC, USA). Data are presented as the mean and standard error (SE). Tukey-Kramer honestly significant difference test was performed, and *p* < 0.05 was considered to the represent statistical significance indicated by the different letters in each figure.

## Results

### BE reduces the accumulation of hypothalamic activated microglia and the gliosis induced by HFD consumption

**Figure 1:**
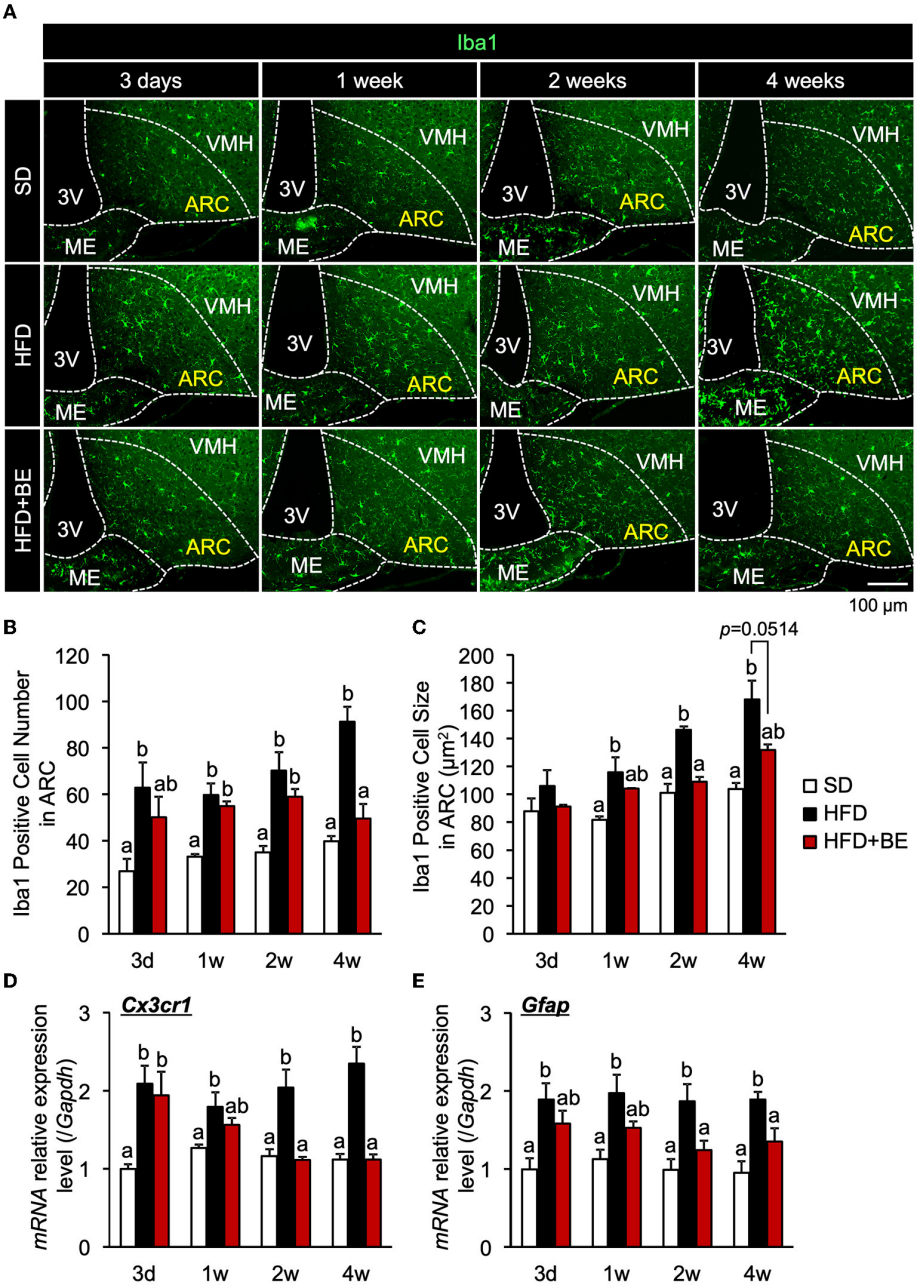
Effects of BE on HFD-induced hypothalamic gliosis in mice. **(A)** Sections of the mediobasal hypothalamus (MBH) of mice (10-μm-thick) were immunofluorescence-stained for Iba1 on Day 3 (3d), Week 1 (1w), Week 2 (2w), and Week 4 (4w) of the feedings. The third ventricle (3V), median eminence (ME), hypothalamic arcuate nucleus (ARC), and ventromedial hypothalamic nucleus (VMH) of the left side of the brain of mice in each group are shown. **(B,C)** Number and size of Iba1-positive cells, respectively, in the ARC. The cell number and size on sections through the ARC of the left and right hemisphere of the brain were measured and mean values calculated. *n* = 3. **(D,E)** Quantitative RT-PCR analysis showing high mRNA expression of gliosis marker, **(D)** *CX3C chemokine receptor 1* (*Cx3cr1*) and **(E)** *Glial fibrillary acidic protein* (*Gfap*), in the whole hypothalamus of mice on Day 3, Week 1, Week 2, and Week 4 of the study. Data shown are mean ± SE (*n* = 5). Different letters represent significant differences among the three groups at each experimental period by Tukey-Kramer honestly significant difference test (*p* < 0.05).

The number of microglia was significantly higher at all of the time points in the HFD-fed group than in the SD-fed group, and the microglia cell size was also significantly larger 2-week after the feedings (Figures 1A–C; Supplementary Figure S1A). These findings are consistent with those of previous studies (9, 10). BE reduced the HFD-induced increases in the number and size of microglia at all the time points (Figures 1A–C; Supplementary Figure S1A). The expression of *CX3C chemokine receptor 1* (*Cx3cr1*; specific to activated microglia) and *Gfap* (specific to activated astrocytes), which is associated with the development of hypothalamic gliosis (9, 10, 15), was significantly higher in the HFD-fed mice than that in the SD-fed mice throughout the experiment (Figures 1D,E). In HFD+BE-fed mice, *Cx3cr1* expression was significantly higher than that in SD-fed mice until day 3, after which it returned to the level of SD-fed mice. In addition, HFD+BE-fed mice showed no significant difference in *Gfap* expression from that of SD-fed mice throughout the feeding period (Figures 1D,E). On the other hand, the visual morphology of GFAP-immunoreactive cells (astrocytes) in ARC and the expression levels of GFAP 4 weeks after the feedings were not different among the three groups (Supplementary Figures S2A,B). These results indicate that BE ameliorates the HFD-induced accumulation of activated microglia and the development of gliosis in the ARC.

### BE reduces pro-inflammatory cytokine gene expression in the hypothalamus

**Figure 2:**
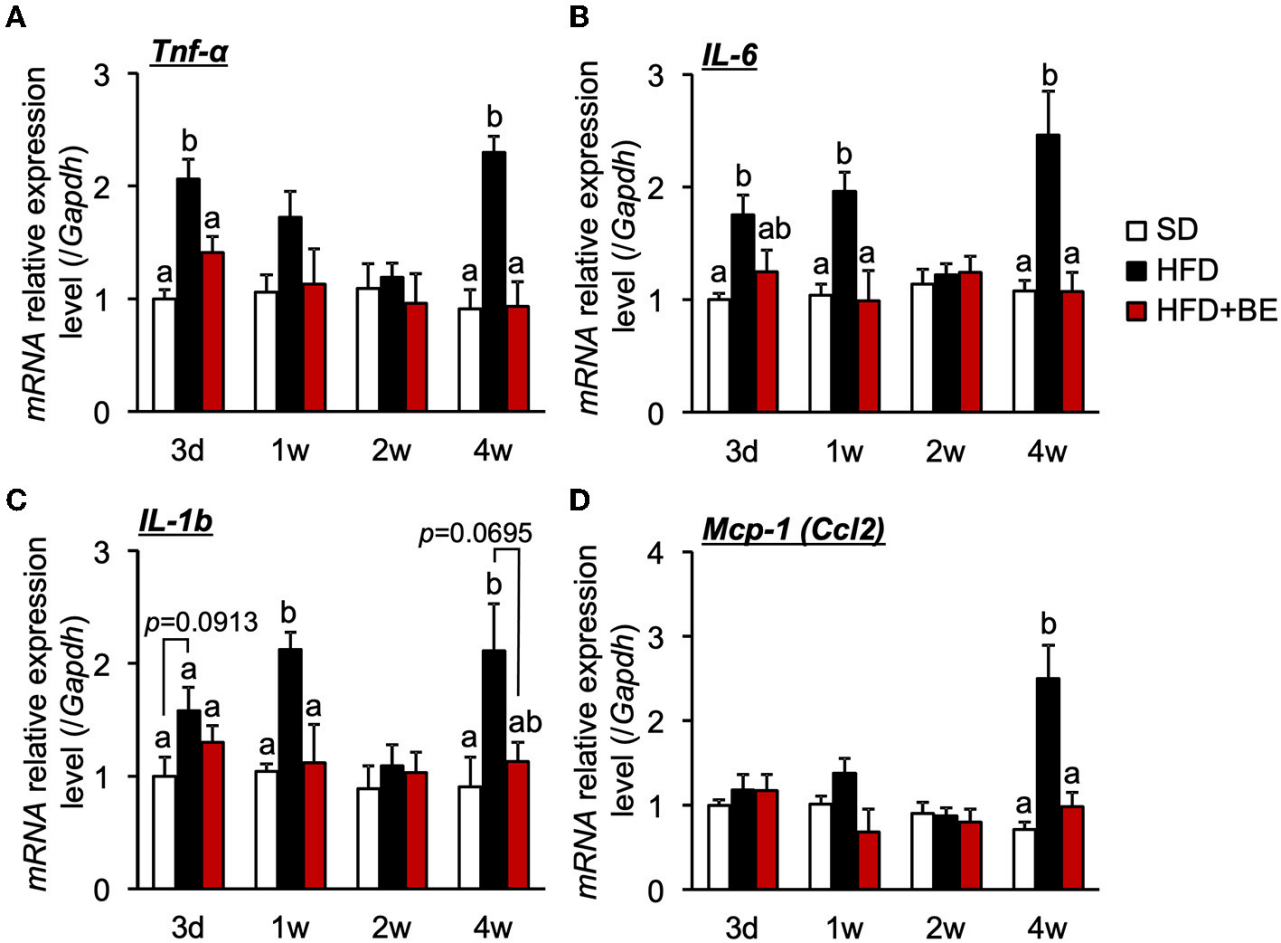
Effects of and HFD and BE on hypothalamic pro-inflammatory cytokine and chemokine gene expression. Quantitative RT-PCR analysis, showing high expression of genes encoding pro-inflammatory cytokines **(A)** *Tnf-*α, **(B)** *Il-6*, **(C)** *Il-1b* and a chemokine **(D)** *Mcp-1* in the whole hypothalamus of mice on Day 3 (3d), Week 1 (1w), Week 2 (2w), and Week 4 (4w) of the feedings. Data shown are mean ± SE (*n* = 5). Different letters represent significant differences among the three groups at each experimental period by Tukey-Kramer honestly significant difference test (*p* < 0.05).

Hypothalamic pro-inflammatory cytokine gene expression correlates with the degree of hypothalamic gliosis (9, 10, 15). Therefore, in the present study, we determined the effect of BE on the expression of genes encoding inflammatory cytokines. We found that HFD-feeding significantly increased the expression of hypothalamic *Tnf-*α on Day 3 (Figure 2A), and that of *Il-6* and *Il-1b* after 1 week (Figures 2B,C). The expression of these mRNAs decreased to the same level as that of SD-fed mice 2 weeks after the feedings, but was again high 4 weeks after the feedings. (Figures 2A–C). BE consumption prevented the HFD-induced increase in pro-inflammatory cytokines expression during the first week and caused a significant reduction in expression vs. the HFD-fed mice at the 4-week time point (Figures 2A–C). BE also prevented the HFD-induced increase in the expression of *Mcp-1* (encoding a chemokine) 4 weeks after the feedings (Figure 2D). Long-term HFD-intake induces inflammation in peripheral, especially visceral, adipose tissue (37). We also found that HFD significantly increased *Il-1b* expression in epididymal fat 4 weeks after the feedings, and supplementation of BE suppressed an increase in the expression of this inflammation marker (Supplementary Figure S3). Contrary, The *Il-1b* expression did not alter on Day 3. These results indicate that BE ameliorates the HFD-caused hypothalamic inflammation induced within 3 days, though it needs long-term to reduce inflammation in peripheral tissues. The inhibition of the acute hypothalamic inflammatory response might have prevented the chronic hypothalamic inflammation that is established 4 weeks after the feedings.

### BE reduces the HFD-induced activation of the NF-κB pathway

**Figure 3:**
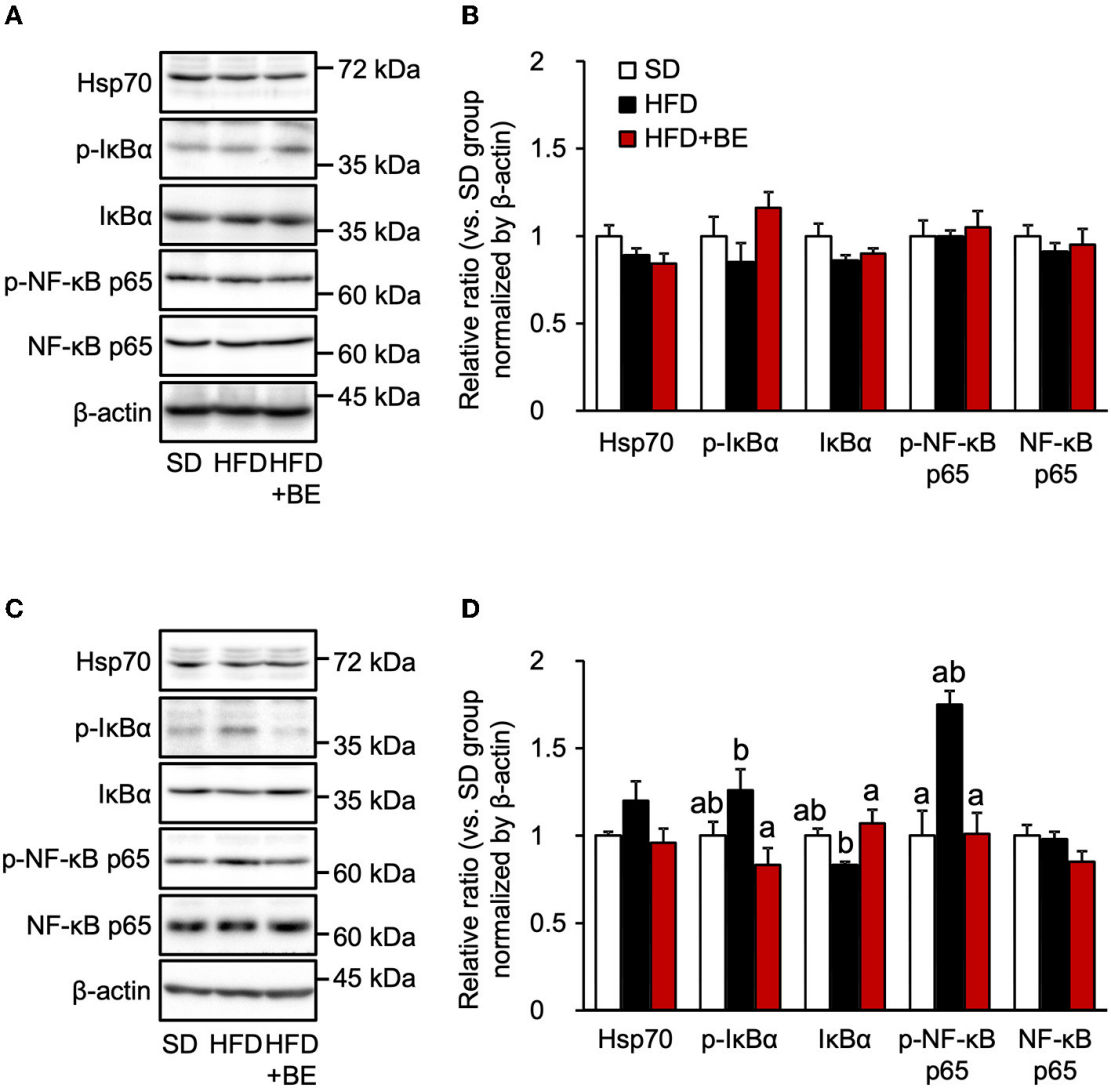
Western blotting analysis of the effects of BE on the expression of proteins involved in the NF-κB pathway. **(A,C)** Expression of 70 kDa heat shock protein (Hsp70), phosphorylated inhibitor of nuclear factor-kappa B alpha (IκBα), IκBα, phosphorylated nuclear factor kappa-B p65 subunit (NF-κB p65), NF-κB p65 and β-actin in the whole hypothalamus extracts of mice on **(A)** Day 3 (3d) and **(C)** Week 4 (4w) of the feedings. **(B,D)** Quantification of the expression levels of proteins in the whole hypothalamic fractions shown in **(A,C)**, respectively. Data shown are mean ± SE (*n* = 5). Different letters represent significant differences among the three groups at each experimental period by Tukey-Kramer honestly significant difference test (*p* < 0.05).

The persistent production of proinflammatory cytokines in the hypothalamus leads to chronic inflammation, characterized by an upregulation of NF-κB transcriptional activity and neuronal stress (12, 13). The nuclear localization signal of NF-κB is usually masked by IκBα, to maintain it in an inactive state, but in response to pro-inflammatory cytokines or other stresses, IκBα is phosphorylated and proteasomally degraded, which permits the phosphorylation in cytoplasm and translocation of NF-κB to the nucleus, where it causes the transcription of genes encoding pro-inflammatory cytokines (38). In the present study, phosphorylation of NF-κB p65 and IκBα was observed 4 weeks after the feedings, though the expression level of IκBα was reduced 4 weeks after the feedings of HFD, as the same time point as the hypothalamic gliosis and inflammation were occurred (Figures 3C,D). Supplementation of BE completely canceled these alterations. The expression of NF-κB and Hsp70, which inhibits NF-κB in response to neuronal stress (39), did not significantly differ among the three groups at any of the time points (Figures 3A–D). These findings imply that the activation NF-κB pathway is involved in the increase in pro-inflammatory cytokine and chemokine gene expression after intake of HFD for long-term, whereas it did not associate with the increase in *Tnf*-α expression observed on Day 3. Thus, the intake of BE quickly reduced the expression of pro-inflammatory cytokine genes, thereby preventing chronic inflammation.

### BE ameliorates the abnormal feeding pattern and obesity induced by HFD consumption

**Figure 4:**
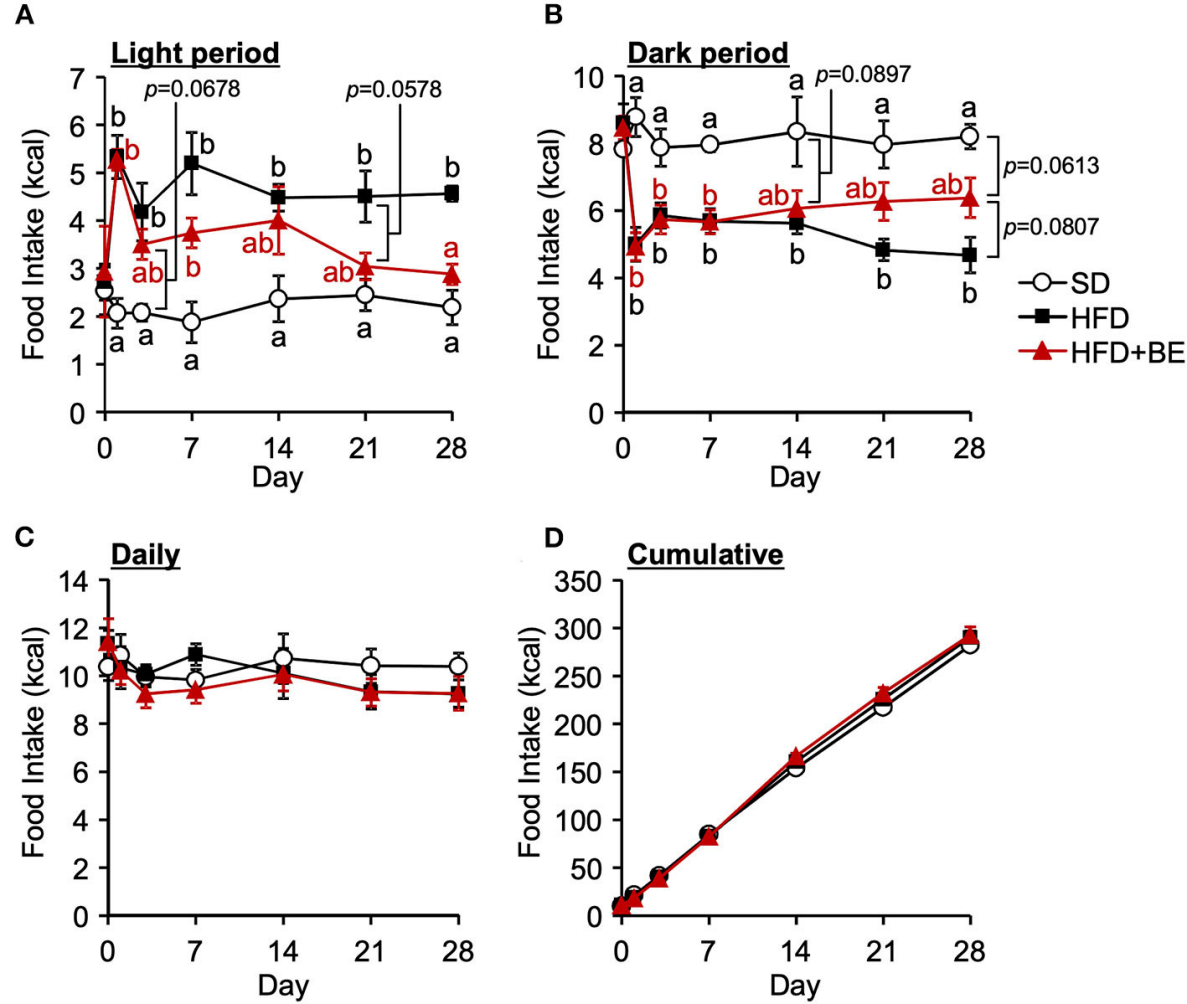
Effects of HFD and BE on the feeding pattern of mice. Food intake during **(A)** the light and **(B)** the dark periods; and **(C)** the daily and **(D)** cumulative food intake during the study period. Data shown are mean ± SE (*n* = 5). Different letters represent significant differences among the three groups by each experimental period by Tukey-Kramer honestly significant difference test (*p* < 0.05).

**Figure 5:**
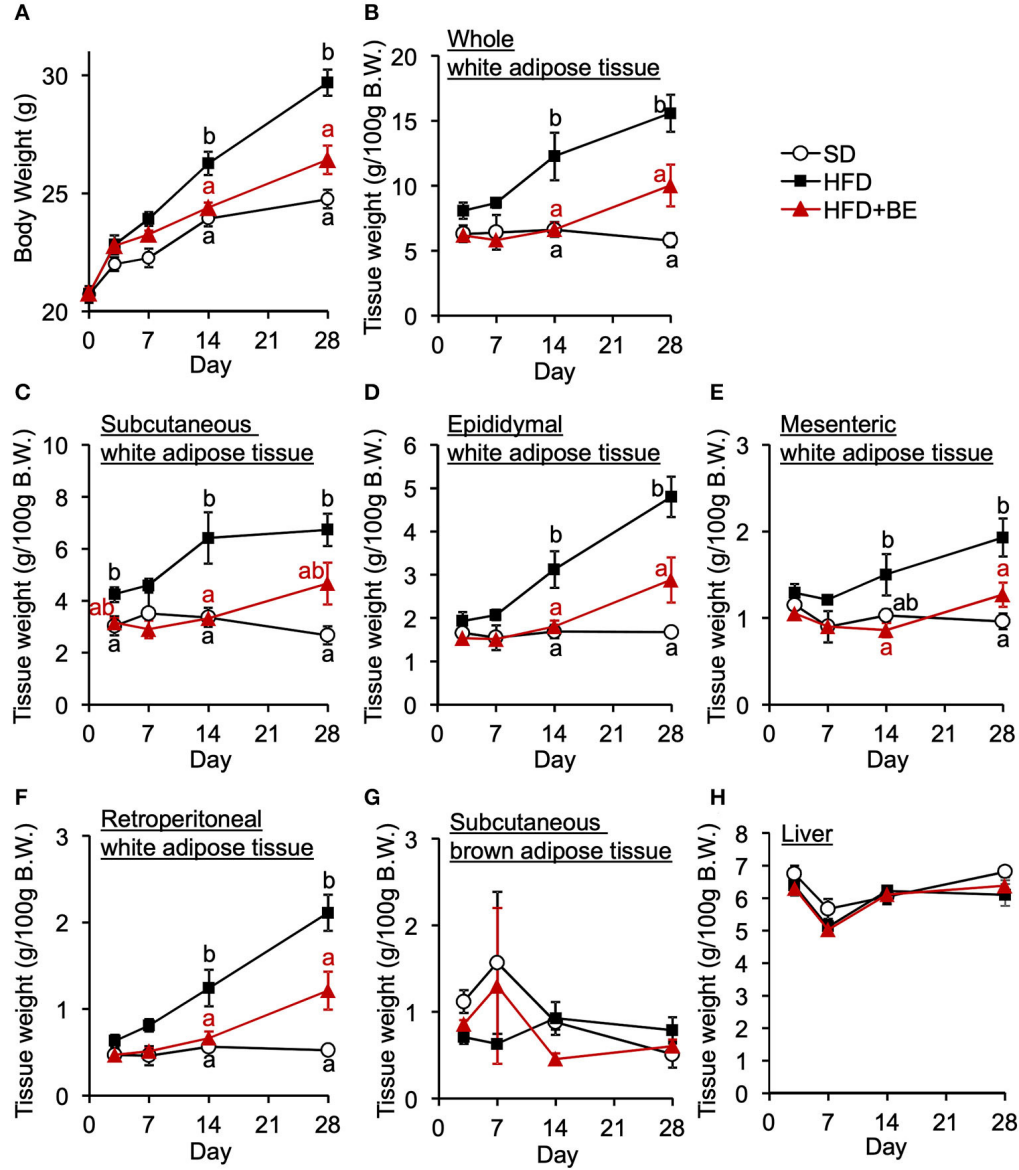
Effects of HFD and BE on the gains in body and fat weight. **(A)** Changes in body weight in each group during the 4-week experimental period. *n* = 8. Weight of **(B)** total white adipose tissue depots, **(C)** subcutaneous white adipose tissue, **(D)** epididymal white adipose tissue, **(E)** mesenteric white adipose tissue, **(F)** retroperitoneal white adipose tissue, and **(G)** subcutaneous brown adipose tissue at each experimental period. *n* = 5. **(H)** Liver weight at each experimental period. *n* = 5. Data shown are mean ± SE. Different letters represent significant differences among the three groups by each experimental period by Tukey-Kramer honestly significant difference test (*p* < 0.05).

Next, we determined whether BE would improve the abnormal feeding pattern. HFD-fed mice significantly increased food intake during the light period and lowered the intake during the dark period from the start of the experiment compared with the SD-fed mice, in which exhibited a robust circadian feeding pattern, with 70–80% of their feeding occurring during the dark period (Figures 4A,B). The BE-fed mice exhibited the same abnormal feeding pattern as the HFD-fed mice until Day 14, but the rhythm was the same as that of the SD group by Day 28 (Figures 4A,B). However, no significant differences were observed in the daily or cumulative food intake among the groups (Figures 4C,D). These differences in feeding pattern were associated with microglial accumulation and the development of gliosis in the ARC. Furthermore, the body and white adipose tissue weights of HFD group were significantly higher between Day 14 and Day 28 of the feedings than those of the SD group. BE inhibited the above deterioration of body composition caused by HFD (Figures 5A–F). However, the brown adipose tissue and liver weight did not differ among the groups (Figures 5G,H). As for plasma lipids, HFD increased the level of non-esterified fatty acids, in particular 1 and 4 weeks after the feedings and BE tended to reduce it; both HFD and HFD+BE decreased the triacylglycerol level; and HFD increased the total cholesterol level 3 days and 4 weeks after the feedings and BE did not lower the HFD-increased total cholesterol level (Supplementary Figure S4). These results suggest that HFD-induced obesity is accompanied by a disruption of the normal circadian feeding pattern, but not necessarily any differences in daily or cumulative energy intake, and that BE ameliorates the effects of an HFD on feeding pattern and prevents obesity.

### C3G reduces the obesity and hypothalamic inflammation induced by HFD consumption

**Figure 6:**
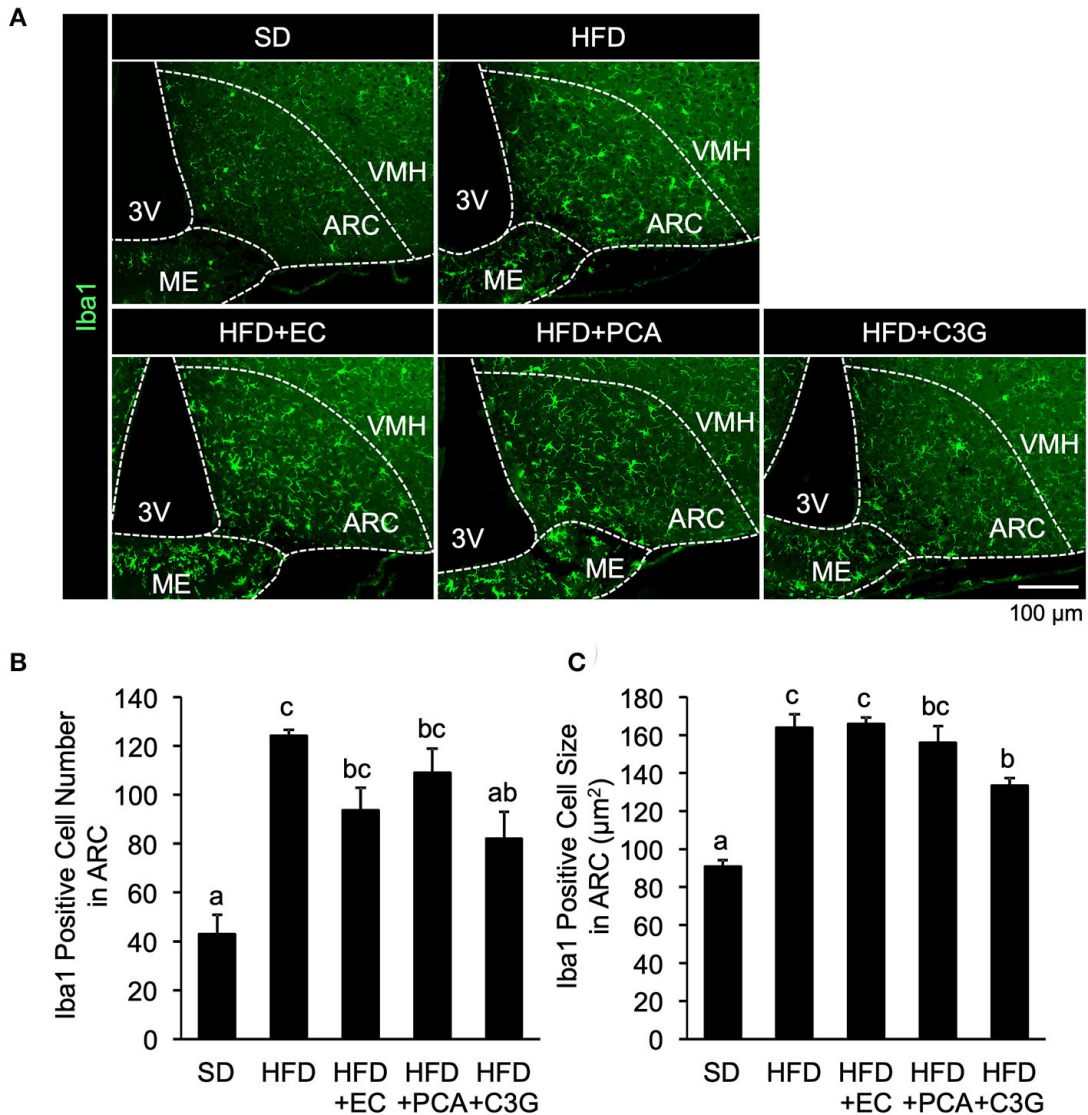
Effects of three fractions derived from BE on the HFD-induced hypothalamic gliosis of mice. **(A)** Sections of the mediobasal hypothalamus (10-μm-thick), immunofluorescence-stained for ionized calcium-binding adapter molecule 1 (Iba1) in mice fed standard diet (SD), high-fat diet (HFD), HFD+EC, HFD+PCA, or HFD+C3G for 4 weeks. The third ventricle (3V), median eminence (ME), hypothalamic arcuate nucleus (ARC), and ventromedial hypothalamic nucleus (VMH) of the left side of the brain of mice from each group are shown. Quantification of **(B)** the number and **(C)** size of Iba1-positive cells in the ARC. The cell number and size in the ARC of the left and right hemispheres of the brain were measured and mean values were calculated. Data shown are mean ± SE (*n* = 3). Different letters represent significant differences among the three groups by each experimental period by Tukey-Kramer honestly significant difference test (*p* < 0.05).

**Figure 7:**
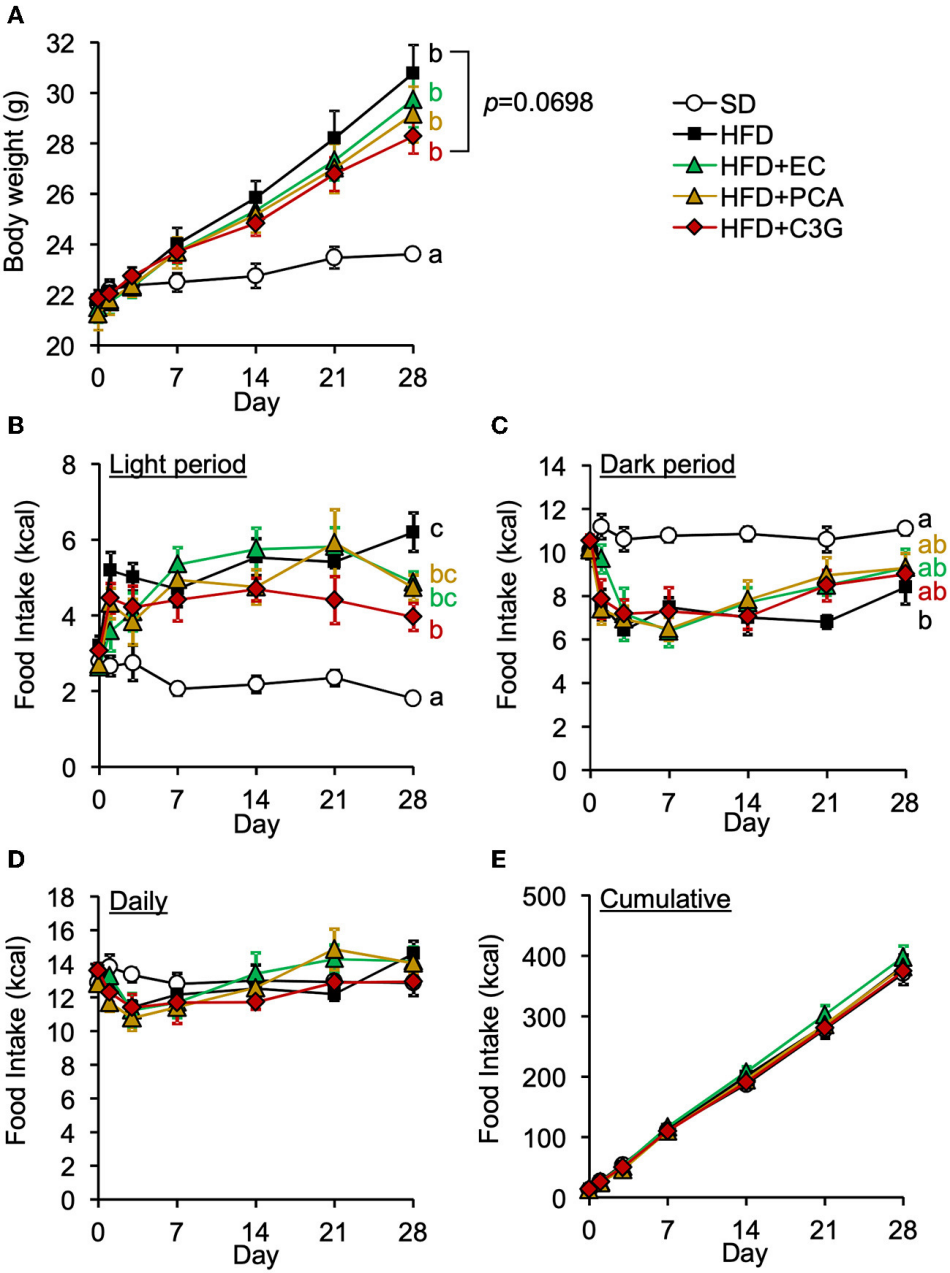
Effects of three fractions derived from BE on the HFD-induced body weight gain and abnormal feeding pattern of mice. **(A)** Changes in body weight in each group during the 4-week study period. Food intake during **(B)** the light and **(C)** dark periods; and **(D)** daily and **(E)** cumulative food intake during the experimental period. Data shown are mean ± SE (*n* = 6). Different letters represent significant differences among the five groups by each experimental period by Tukey-Kramer honestly significant difference test (*p* < 0.05). The results of statistical analysis are shown only at the end points and the statistical data for the other time points are listed in Supplementary Table S2.

We next attempted to identify the active compound in BE that is responsible for its beneficial effects on the HFD-induced hypothalamic inflammation, abnormality in feeding pattern, and obesity. The EC, PCA, and C3G fractions were prepared from BE and separately given to mice for 4 weeks. Of these, only C3G significantly reduced the HFD-induced hypothalamic microglial accumulation (Figures 6A-C; Supplementary Figure S1B). C3G also tended to reduce the body weight gain induced by HFD consumption (Figure 7A) and reduced the light period hyperphagia (Figure 7B). The consumption of the EC or PCA fractions failed to prevent the weight gain or the abnormal feeding pattern induced by the HFD (Figures 7A–C). No differences were found in the daily or cumulative food intake among the groups (Figures 7D,E). These results suggest that C3G is the active compound in BE that is principally responsible for the prevention of the chronic inflammation in the ARC induced by HFD consumption and that it reduces obesity by preventing the effects of HFD on the circadian feeding pattern.

## Discussion

The consumption of HFD causes obesity not only through high calorie intake but also by disturbing the normal feeding pattern. HFD consumption has been reported to rapidly induce a disruption in feeding rhythm, which is accompanied by hypothalamic inflammation, before significant increases in body or adipose tissue weight (11). Because these acute pathological defects, i.e., feeding rhythm disruption and hypothalamic inflammation, likely precede systemic metabolic disruption, suppression of pathological defects may represent an effective approach to the prevention of DIO. In the present study, we evaluated the effects of BE on the abnormal feeding pattern and hypothalamic inflammation induced by HFD-feeding, which precede HFD-induced obesity. We found that BE reduces HFD-caused pro-inflammatory cytokines expression (Figure 2) and prevents the development of chronic inflammation, characterized by activation of the NF-κB pathway 4 weeks after the feedings (Figure 3). BE ingestion reduces HFD-induced activated microglial accumulation in the ARC (Figure 1) and restores the normal feeding pattern (Figure 4), thereby preventing the marked weight gain induced by HFD (Figure 5). In addition, we have shown that C3G is an active compound in BE and principally mediates these effects (Figures 6, 7). This is the first study to show that a functional food material has an anti-obesity effect by ameliorating the hypothalamic inflammation and restoring the abnormal feeding pattern associated with DIO. Therefore, the results of the present study provide new insight into the anti-obesity effects of BE and its components.

HFD-induced hypothalamic inflammation impairs normal neurotransmission, resulting in a disruption to feeding rhythm (11). Within a week of HFD intake, the activation of microglia disrupts neurotransmission, in the form of excessive glutamate release from gap-junction hemichannels on the cell surface (40), and this activation lead to the activation of microglia and astrocytes accompanied by the release pro-inflammatory cytokines (9, 10), which recruit peripheral myeloid cells to the ARC, leading to chronic inflammation, characterized by the activation of the NF-κB pathway (10, 13) and persistent neurological dysfunction, and subsequent disruption of the feeding rhythm and induction of obesity. In the present study, BE and C3G had beneficial effects on the abnormal feeding pattern induced by HFD from 2 weeks (Figures 4, 7). The inhibition of neurotransmission *via* the microglial gap-junction hemichannel pathway is transient (11, 40), occurring within the first few days of HFD-consumption. This suggests that BE does not reduce the activation of the microglial gap junction hemichannel pathway. However, BE attenuated the HFD-induced increase in hypothalamic pro-inflammatory cytokine gene expression at all of the time points during the feeding periods (Figure 2). This implies that the inhibition of inflammatory cytokine signaling is involved in the amelioration of the HFD-induced abnormality in feeding pattern by BE and C3G.

The decline in the neurological function of the hypothalamus that is caused by HFD-induced inflammation (9, 13) induces a loss of feeding rhythm and obesity (41, 42). In the present study, BE consumption attenuated the reduction in hypothalamic *Pro-opiomelanocortin* (*Pomc)* expression caused 4 weeks after HFD-intake (Supplementary Figure S5). In the ARC, anorexigenic agouti-related peptide/neuro peptide Y (AgRP/NPY)-expressing neurons and orexigenic POMC/cocaine- and amphetamine-regulated transcript (CART)-expressing neurons sense systemic metabolic status by monitoring the concentrations of hormones and nutrients, delivered *via* the circulation or visceral sensory nerves, and precisely regulate feeding behavior (43). Recent studies using the designer receptor exclusively activated by designer drugs (also known as DREADD) system have shown that HFD consumption reduces the responsiveness of POMC- and AgRP-expressing neurons (44–46). In addition, chronic HFD consumption has been shown to induce the specific apoptosis of POMC-expressing neurons in the ARC (10). These neurons regulate feeding behavior and peripheral metabolism in response to leptin (47, 48), a satiety hormone that is released by adipose tissue. We found that BE ameliorates the loss of leptin sensitivity in the ARC that is induced by an HFD (data not shown). These findings may imply that BE ameliorates the neuronal hypersensitivity, abnormal feeding pattern, and systemic metabolic dysfunction through the suppression of hypothalamic inflammation.

Sustained abnormalities in feeding pattern ultimately disrupt the circadian rhythms of peripheral organs, causing metabolic dysfunction. The circadian rhythm of the suprachiasmatic nucleus (SCN) of the hypothalamus, the central clock that controls the systemic circadian rhythm, is reset by light arriving at the retina and is not affected by feeding. However, the feeding rhythm is disrupted within a day of the start of HFD consumption, which is earlier than disruptions occur in peripheral organs. This implies that the feeding rhythm is more strongly influenced by meals than the SCN and that the disruption of the feeding rhythm underpins HFD-induced obesity. In the present study, the total energy intake of each group was similar (Figures 4D, 7E), but only the HFD-fed mice, which exhibited an abnormal feeding pattern, showed significant weight gain (Figures 4A,B, 7B,C). Previous studies have shown that oral administration of D-allulose or pectin-containing carbonated water ameliorates obesity in mice through suppressing HFD-caused light period hyperphagia (49, 50). In fact, previous studies have shown that the restriction of HFD intake during the light phase alone prevents obesity and disruption to the circadian rhythm in peripheral tissues (3, 51, 52). These findings suggest that the keeping of an appropriate feeding rhythm is essential for the maintenance of metabolic homeostasis and the circadian rhythm.

In the present study, we found that C3G was the most effective compound for preventing HFD-induced hypothalamic inflammation and feeding rhythm disruption. The principal mechanism underpinning the anti-inflammatory effect of C3G has previously been shown to be a reduction in the oxidative stress induced by lipopolysaccharide (LPS) or saturated long-chain fatty acids, upstream of the NF-κB pathway (53, 54). C3G and its metabolites have been reported to inhibit the LPS- or saturated long-chain fatty acids-induced activation of NF-κB pathway and expression of pro-inflammatory cytokines in the cultured microglial cells (24, 55–60) and the cells in peripheral tissues. Furthermore, the intake of C3G-rich meals decreases the expression level of pro-inflammatory cytokines in the peripheral tissues of mice with DIO (59, 61). One possible mechanism for the inhibitory effect of C3G on hypothalamic inflammation is that C3G or its metabolites enter the hypothalamus and possess direct antioxidant effects. However, orally administered C3G is quickly metabolized to cyanidin, protocatechuic acid, phenolic acids, and phloroglucinol aldehyde, and only ~12.4% is absorbed in its intact form (23, 62, 63). Nevertheless, C3G and its metabolites have been detected in various organs, including the hypothalamus (64). In addition, when rats feed C3G-enriched diets for ~2 weeks, the concentrations of C3G and its metabolites in the brain are higher than those in the plasma (64, 65), suggesting that C3G and its metabolites may accumulate in the brain. Because C3G and its metabolites have been shown to be able to cross the blood-brain barrier (BBB) (62) and part of the ARC lacking area of BBB (66), these compounds may be able to access the ARC *via* the circulation. However, these previous findings are not enough to explain the anti-inflammatory effects of C3G, because C3G consumption also affects hormone secretion and the gut microbiota (67). Further characterization of the pharmacokinetics and other aspects of these substances should help clarify the mechanism by which C3G ameliorates inflammation *in vivo*. In the present study, we did not address to the detailed anti-inflammatory mechanism and pharmacokinetics of C3G and further study is needed in future.

In the present study, we found that C3G alone could not suppress the weight gain or correct the feeding rhythm disturbance as same as BE did. One possible explanation for this is that the duration of C3G consumption was too short to have an effect. In other studies, HFD-induced weight gain was significantly reduced by 8–16 weeks of ingestion of C3G through supplementation to the diet (68, 69), or the drinking water (70, 71), and orally administration (72). Thus, we should investigate absorption and accumulation of C3G and its metabolites and estimate whether concentration is enough to prevent DIO in future. It is also possible that the C3G and flavan 3-ols in BE have additive or synergistic effects to prevent DIO. Previous studies have shown that the oral administration of B-type PCAs promotes heat production *via* brain-peripheral organ axes (73, 74). Therefore, the suppression of hypothalamic inflammation by C3G may be coordinate with falvan 3-ols to improve metabolic function *via* the neural axis, resulting in a significant suppression of DIO by BE. However, further study is needed to evaluate this issue.

In conclusion, we have demonstrated that BE ingestion reduces HFD-induced hypothalamic inflammation, resulting in amelioration of the abnormal feeding pattern and DIO. Many researchers gradually pay an attention that the CNS is a therapeutic target for DIO, and the effect of functional foods on the CNS is a growing area of interest. The results from the present study indicate that BE is an attractive functional food material for regulating the CNS function and provide new insights into the mechanism by which it prevents obesity, in particular DIO.

## Data availability statement

The original contributions presented in the study are included in the article/Supplementary files, further inquiries can be directed to the corresponding authors.

## Ethics statement

The animal study was reviewed and approved by Institutional Animal Care and Use Committee in Kobe University (permission number: 2020-10-13). Written informed consent was obtained from the owners for the participation of their animals in this study.

## Author contributions

K-yH, YY, and HA designed and conceived the experiments and wrote the manuscript. K-yH performed the experiments. All authors contributed to the data interpretation, drafting of the manuscript, manuscript writing, and approved the submitted version.

## Funding

This study was supported by a Grant-in-Aid for Scientific Research from the Japan Society for the Promotion of Science (number 21J20240).

## Conflict of interest

The authors declare that the research was conducted in the absence of any commercial or financial relationships that could be construed as a potential conflict of interest.

## Publisher's note

All claims expressed in this article are solely those of the authors and do not necessarily represent those of their affiliated organizations, or those of the publisher, the editors and the reviewers. Any product that may be evaluated in this article, or claim that may be made by its manufacturer, is not guaranteed or endorsed by the publisher.

## Abbreviations

3V: third ventricle
ARC: arcuate nucleus of the hypothalamus
AgRP: agouti-related protein
BBB: blood brain barrier
BE: black soybean seed coat (polyphenol-rich) extract
C3G: cyanidin 3-*O*-glucoside
CART: cocaine- and amphetamine-regulated transcript
CNS: central nervous system
Cx3cr1: CX3C-motif chemokine receptor 1
DIO: diet induced obesity
EC: epicatechin and catechin
Gapdh: glyceraldehyde-3-phosphate dehydrogenase
Gfap: glial fibrillary acidic protein
HFD: high fat diet
Hsp70: 70kDa heat shock protein
Iba1: ionized calcium-binding adapter molecule 1
Il-1b: interleukin 1 beta
Il-6: interleukin 6
IκBα: inhibitor of nuclear factor-kappa B alpha
LPS: Lipopolysaccharides
MBH: mediobasal hypothalamus
Mcp1: monocyte chemotactic protein 1
ME: median eminence
NF-κB: nuclear factor-kappa B
NPY: neuropeptide Y
PCA: procyanidin
POMC: Pro-opiomelanocortin
SCN: suprachiasmatic nucleus
SD: standard diet
Tnf-α: Tumor necrosis factor alpha
VMH: ventromedial hypothalamic nucleus.
