# Supplementary material for: Black soybean seed coat polyphenol ameliorates the abnormal feeding pattern induced by high-fat diet consumption
Source: Front Nutr. 2022 Oct 10;9:1006132. doi: 10.3389/fnut.2022.1006132 (PMC9589235; doi:10.3389/fnut.2022.1006132)
Supplement: Supplementary file 1 [file Data_Sheet_1.docx]

Supplementary materials

**Supplementary Table 1. Primer sequences used for qRT-PCR**

**Gene Sequence (5’- -3’)**

*Gapdh* Forward: CATGGCCTTCCGTGTTCCTA
 Reverse: CCTGCTTCACCACCTTCTTGA

*Tnf-α* Forward: GACAGTGACCTGGACTGTGG
 Reverse: TGAGACAGAGGCAACCTGAC

*Il-1b* Forward: GTTGACGGACCCCAAAAGATG
 Reverse: GCTGCTGCGAGATTTGAAGC

*Il-6* Forward: AGTCCGGAGAGGAGACTTCA
 Reverse: ATTTCCACGATTTCCCAGAG

*Ccl2* Forward: AGGTCCCATGTCATGCTTCTGG
 Reverse: CTGCTGCTGGTGATCCTCTTG

*Cx3cr1* Forward: ACCTCCTTCCCTGAACTGGA
 Reverse: AGACCGAACGTGAAGACGAG

*Gfap* Forward: GTTTCATCTTGGAGCTTCTGC
 Reverse: GGAGGTGGAGAGGGACAAC

*Pomc* Forward: GACACGTGGAAGATGCCGAG
 Reverse: CAGCGAGAGGTCGAGTTTGC

*Cart* Forward: CGAGAAGAAGTACGGCCAAG
 Reverse: CACACAGCTTCCCGATCC

*Agrp* Forward: GAGTTCCCAGGTCTAAGTCTGAATG
 Reverse: ATCTAGCACCTCCGCCAAAG

*Npy* Forward: TCAGACCTCTTAATGAAGGAAAGCA
 Reverse: ATGAGGGTGGAAACTTGGAAAAG

*Orexin* Forward: TTTGGACCACTGCACTGAAGA
 Reverse: CCAGGGAACCTTTGTAGAAGGAA

*Mch* Forward: ACTGCAGAAAGATCCGTTGTC
 Reverse: GTTTGGAGCCTGTGTTCTTTG

**Supplementary Table 2. Detailed statistical analysis data for Figure 7.**

| **Figure 7A** |  |  |  |  |  |  |  |  |  |  |  |  |
| --- | --- | --- | --- | --- | --- | --- | --- | --- | --- | --- | --- | --- |
|  |  |  |  |  |  |  |  |  |  |  |  |  |
| **Figure 7A (Day 0)** | |  |  |  |  |  | **Figure 7A (Day 14)** | |  |  |  |  |
| Group | Significance | Average |  |  |  |  | Group | Significance | Average |  |  |  |
| SD | A | 21.616667 |  |  |  |  | SD | A | 22.726667 |  |  |  |
| HFD | A | 21.776667 |  |  |  |  | HFD | B | 25.823333 |  |  |  |
| HFD+EC | A | 21.46 |  |  |  |  | HFD+EC | B | 25.316667 |  |  |  |
| HFD+PCA | A | 21.213333 |  |  |  |  | HFD+PCA | AB | 25.15 |  |  |  |
| HFD+C3G | A | 21.843333 |  |  |  |  | HFD+C3G | AB | 24.823333 |  |  |  |
|  |  |  |  |  |  |  |  |  |  |  |  |  |
| P value (vs.) | SD | HFD | HFD+EC | HFD+PCA | HFD+C3G |  | P value (vs.) | SD | HFD | HFD+EC | HFD+PCA | HFD+C3G |
| SD | – | – | – | – | – |  | SD | – | – | – | – | – |
| HFD | 0.999 | – | – | – | – |  | HFD | 0.0096 | – | – | – | – |
| HFD+EC | 0.999 | 0.9857 | – | – | – |  | HFD+EC | 0.0385 | 0.9741 | – | – | – |
| HFD+PCA | 0.9654 | 0.8921 | 0.9944 | – | – |  | HFD+PCA | 0.0591 | 0.9298 | 0.9996 | – | – |
| HFD+C3G | 0.996 | 1 | 0.9712 | 0.8472 | – |  | HFD+C3G | 0.129 | 0.7624 | 0.9765 | 0.995 | – |
|  |  |  |  |  |  |  |  |  |  |  |  |  |
| **Figure 7A (Day 1)** | |  |  |  |  |  | **Figure 7A (Day 21)** | |  |  |  |  |
| Group | Significance | Average |  |  |  |  | Group | Significance | Average |  |  |  |
| SD | A | 22.19 |  |  |  |  | SD | A | 23.456667 |  |  |  |
| HFD | A | 22.126667 |  |  |  |  | HFD | B | 28.176667 |  |  |  |
| HFD+EC | A | 21.7 |  |  |  |  | HFD+EC | B | 27.303333 |  |  |  |
| HFD+PCA | A | 21.783333 |  |  |  |  | HFD+PCA | B | 26.996667 |  |  |  |
| HFD+C3G | A | 22.016667 |  |  |  |  | HFD+C3G | AB | 26.766667 |  |  |  |
|  |  |  |  |  |  |  |  |  |  |  |  |  |
| P value (vs.) | SD | HFD | HFD+EC | HFD+PCA | HFD+C3G |  | P value (vs.) | SD | HFD | HFD+EC | HFD+PCA | HFD+C3G |
| SD | – | – | – | – | – |  | SD | – | – | – | – | – |
| HFD | 1 | – | – | – | – |  | HFD | 0.0035 | – | – | – | – |
| HFD+EC | 0.9237 | 0.9525 | – | – | – |  | HFD+EC | 0.0213 | 0.9414 | – | – | – |
| HFD+PCA | 0.9598 | 0.9782 | 0.9999 | – | – |  | HFD+PCA | 0.0387 | 0.8448 | 0.9988 | – | – |
| HFD+C3G | 0.9984 | 0.9997 | 0.9838 | 0.9949 | – |  | HFD+C3G | 0.0596 | 0.7422 | 0.99 | 0.9996 | – |
|  |  |  |  |  |  |  |  |  |  |  |  |  |
| **Figure 7A (Day 3)** | |  |  |  |  |  | **Figure 7A (Day 28)** | |  |  |  |  |
| Group | Significance | Average |  |  |  |  | Group | Significance | Average |  |  |  |
| SD | A | 22.37 |  |  |  |  | SD | A | 23.608 |  |  |  |
| HFD | A | 22.553333 |  |  |  |  | HFD | B | 30.75 |  |  |  |
| HFD+EC | A | 22.3 |  |  |  |  | HFD+EC | B | 29.723333 |  |  |  |
| HFD+PCA | A | 22.353333 |  |  |  |  | HFD+PCA | B | 30.048 |  |  |  |
| HFD+C3G | A | 22.716667 |  |  |  |  | HFD+C3G | B | 28.26 |  |  |  |
|  |  |  |  |  |  |  |  |  |  |  |  |  |
| P value (vs.) | SD | HFD | HFD+EC | HFD+PCA | HFD+C3G |  | P value (vs.) | SD | HFD | HFD+EC | HFD+PCA | HFD+C3G |
| SD | – | – | – | – | – |  | SD | – | – | – | – | – |
| HFD | 0.9973 | – | – | – | – |  | HFD | <.0001 | – | – | – | – |
| HFD+EC | 0.9999 | 0.9906 | – | – | – |  | HFD+EC | 0.0007 | 0.9139 | – | – | – |
| HFD+PCA | 1 | 0.9962 | 1 | – | – |  | HFD+PCA | 0.0006 | 0.9808 | 0.999 | – | – |
| HFD+C3G | 0.9701 | 0.9983 | 0.9428 | 0.9646 | – |  | HFD+C3G | 0.0107 | 0.2765 | 0.7498 | 0.633 | – |
|  |  |  |  |  |  |  |  |  |  |  |  |  |
| **Figure 7A (Day 7)** | |  |  |  |  |  |  |  |  |  |  |  |
| Group | Significance | Average |  |  |  |  |  |  |  |  |  |  |
| SD | A | 22.486667 |  |  |  |  |  |  |  |  |  |  |
| HFD | A | 24.003333 |  |  |  |  |  |  |  |  |  |  |
| HFD+EC | A | 23.686667 |  |  |  |  |  |  |  |  |  |  |
| HFD+PCA | A | 23.66 |  |  |  |  |  |  |  |  |  |  |
| HFD+C3G | A | 23.693333 |  |  |  |  |  |  |  |  |  |  |
|  |  |  |  |  |  |  |  |  |  |  |  |  |
| P value (vs.) | SD | HFD | HFD+EC | HFD+PCA | HFD+C3G |  |  |  |  |  |  |  |
| SD | – | – | – | – | – |  |  |  |  |  |  |  |
| HFD | 0.2611 | – | – | – | – |  |  |  |  |  |  |  |
| HFD+EC | 0.4853 | 0.9922 | – | – | – |  |  |  |  |  |  |  |
| HFD+PCA | 0.5071 | 0.9894 | 1 | – | – |  |  |  |  |  |  |  |
| HFD+C3G | 0.4799 | 0.9928 | 1 | 1 | – |  |  |  |  |  |  |  |

| **Figure 7B** |  |  |  |  |  |  |  |  |  |  |  |  |
| --- | --- | --- | --- | --- | --- | --- | --- | --- | --- | --- | --- | --- |
|  |  |  |  |  |  |  |  |  |  |  |  |  |
| **Figure 7B (Day 0)** | |  |  |  |  |  | **Figure 7B (Day 14)** | |  |  |  |  |
| Group | Significance | Average |  |  |  |  | Group | Significance | Average |  |  |  |
| SD | A | 12.89 |  |  |  |  | SD | A | 187.48167 |  |  |  |
| HFD | A | 13.556667 |  |  |  |  | HFD | A | 200.765 |  |  |  |
| HFD+EC | A | 12.845 |  |  |  |  | HFD+EC | A | 207.33833 |  |  |  |
| HFD+PCA | A | 12.803333 |  |  |  |  | HFD+PCA | A | 194.52167 |  |  |  |
| HFD+C3G | A | 13.59 |  |  |  |  | HFD+C3G | A | 190.95667 |  |  |  |
|  |  |  |  |  |  |  |  |  |  |  |  |  |
| P value (vs.) | SD | HFD | HFD+EC | HFD+PCA | HFD+C3G |  | P value (vs.) | SD | HFD | HFD+EC | HFD+PCA | HFD+C3G |
| SD | – | – | – | – | – |  | SD | – | – | – | – | – |
| HFD | 0.6355 | – | – | – | – |  | HFD | 0.6091 | – | – | – | – |
| HFD+EC | 1 | 0.5777 | – | – | – |  | HFD+EC | 0.2306 | 0.9517 | – | – | – |
| HFD+PCA | 0.9997 | 0.5244 | 1 | – | – |  | HFD+PCA | 0.9388 | 0.9597 | 0.6401 | – | – |
| HFD+C3G | 0.5927 | 1 | 0.535 | 0.4826 | – |  | HFD+C3G | 0.9954 | 0.8234 | 0.4094 | 0.995 | – |
|  |  |  |  |  |  |  |  |  |  |  |  |  |
| **Figure 7B (Day 1)** | |  |  |  |  |  | **Figure 7B (Day 21)** | |  |  |  |  |
| Group | Significance | Average |  |  |  |  | Group | Significance | Average |  |  |  |
| SD | A | 26.711667 |  |  |  |  | SD | A | 278.47667 |  |  |  |
| HFD | A | 26.308333 |  |  |  |  | HFD | A | 285.115 |  |  |  |
| HFD+EC | A | 26.003333 |  |  |  |  | HFD+EC | A | 302.61333 |  |  |  |
| HFD+PCA | A | 24.36 |  |  |  |  | HFD+PCA | A | 286.05833 |  |  |  |
| HFD+C3G | A | 25.751667 |  |  |  |  | HFD+C3G | A | 280.56333 |  |  |  |
|  |  |  |  |  |  |  |  |  |  |  |  |  |
| P value (vs.) | SD | HFD | HFD+EC | HFD+PCA | HFD+C3G |  | P value (vs.) | SD | HFD | HFD+EC | HFD+PCA | HFD+C3G |
| SD | – | – | – | – | – |  | SD | – | – | – | – | – |
| HFD | 0.9878 | – | – | – | – |  | HFD | 0.9921 | – | – | – | – |
| HFD+EC | 0.9097 | 0.9958 | – | – | – |  | HFD+EC | 0.5213 | 0.7799 | – | – | – |
| HFD+PCA | 0.0609 | 0.1607 | 0.3006 | – | – |  | HFD+PCA | 0.9869 | 1 | 0.812 | – | – |
| HFD+C3G | 0.7726 | 0.9604 | 0.998 | 0.4615 | – |  | HFD+C3G | 0.9999 | 0.9981 | 0.605 | 0.9961 | – |
|  |  |  |  |  |  |  |  |  |  |  |  |  |
| **Figure 7B (Day 3)** | |  |  |  |  |  | **Figure 7B (Day 28)** | |  |  |  |  |
| Group | Significance | Average |  |  |  |  | Group | Significance | Average |  |  |  |
| SD | A | 54.413333 |  |  |  |  | SD | A | 373.28 |  |  |  |
| HFD | AB | 50.605 |  |  |  |  | HFD | A | 383.53833 |  |  |  |
| HFD+EC | AB | 51.533333 |  |  |  |  | HFD+EC | A | 397.92 |  |  |  |
| HFD+PCA | B | 45.703333 |  |  |  |  | HFD+PCA | A | 389.78 |  |  |  |
| HFD+C3G | AB | 49.801667 |  |  |  |  | HFD+C3G | A | 374.76167 |  |  |  |
|  |  |  |  |  |  |  |  |  |  |  |  |  |
| P value (vs.) | SD | HFD | HFD+EC | HFD+PCA | HFD+C3G |  | P value (vs.) | SD | HFD | HFD+EC | HFD+PCA | HFD+C3G |
| SD | – | – | – | – | – |  | SD | – | – | – | – | – |
| HFD | 0.5543 | – | – | – | – |  | HFD | 0.9858 | – | – | – | – |
| HFD+EC | 0.7754 | 0.9956 | – | – | – |  | HFD+EC | 0.7404 | 0.943 | – | – | – |
| HFD+PCA | 0.014 | 0.31 | 0.1656 | – | – |  | HFD+PCA | 0.9333 | 0.9979 | 0.9941 | – | – |
| HFD+C3G | 0.3683 | 0.9975 | 0.9557 | 0.484 | – |  | HFD+C3G | 1 | 0.9905 | 0.7501 | 0.9439 | – |
|  |  |  |  |  |  |  |  |  |  |  |  |  |
| **Figure 7B (Day 7)** | |  |  |  |  |  |  |  |  |  |  |  |
| Group | Significance | Average |  |  |  |  |  |  |  |  |  |  |
| SD | A | 111.70333 |  |  |  |  |  |  |  |  |  |  |
| HFD | A | 112.12167 |  |  |  |  |  |  |  |  |  |  |
| HFD+EC | A | 116.63667 |  |  |  |  |  |  |  |  |  |  |
| HFD+PCA | A | 109.03333 |  |  |  |  |  |  |  |  |  |  |
| HFD+C3G | A | 109.76167 |  |  |  |  |  |  |  |  |  |  |
|  |  |  |  |  |  |  |  |  |  |  |  |  |
| P value (vs.) | SD | HFD | HFD+EC | HFD+PCA | HFD+C3G |  |  |  |  |  |  |  |
| SD | – | – | – | – | – |  |  |  |  |  |  |  |
| HFD | 1 | – | – | – | – |  |  |  |  |  |  |  |
| HFD+EC | 0.858 | 0.8924 | – | – | – |  |  |  |  |  |  |  |
| HFD+PCA | 0.9828 | 0.9707 | 0.5576 | – | – |  |  |  |  |  |  |  |
| HFD+C3G | 0.9948 | 0.9891 | 0.647 | 0.9999 | – |  |  |  |  |  |  |  |

| **Figure 7C** |  |  |  |  |  |  |  |  |  |  |  |  |
| --- | --- | --- | --- | --- | --- | --- | --- | --- | --- | --- | --- | --- |
|  |  |  |  |  |  |  |  |  |  |  |  |  |
| **Figure 7C (Day 0)** | |  |  |  |  |  | **Figure 7C (Day 14)** | |  |  |  |  |
| Group | Significance | Average |  |  |  |  | Group | Significance | Average |  |  |  |
| SD | A | 12.888333 |  |  |  |  | SD | A | 13.025 |  |  |  |
| HFD | A | 13.563333 |  |  |  |  | HFD | A | 12.541667 |  |  |  |
| HFD+EC | A | 12.85 |  |  |  |  | HFD+EC | A | 13.433333 |  |  |  |
| HFD+PCA | A | 12.8 |  |  |  |  | HFD+PCA | A | 12.565 |  |  |  |
| HFD+C3G | A | 13.591667 |  |  |  |  | HFD+C3G | A | 11.731667 |  |  |  |
|  |  |  |  |  |  |  |  |  |  |  |  |  |
| P value (vs.) | SD | HFD | HFD+EC | HFD+PCA | HFD+C3G |  | P value (vs.) | SD | HFD | HFD+EC | HFD+PCA | HFD+C3G |
| SD | – | – | – | – | – |  | SD | – | – | – | – | – |
| HFD | 0.6238 | – | – | – | – |  | HFD | 0.9912 | – | – | – | – |
| HFD+EC | 1 | 0.5744 | – | – | – |  | HFD+EC | 0.9954 | 0.92 | – | – | – |
| HFD+PCA | 0.9997 | 0.5106 | 1 | – | – |  | HFD+PCA | 0.9927 | 1 | 0.9268 | – | – |
| HFD+C3G | 0.5873 | 1 | 0.5381 | 0.4752 | – |  | HFD+C3G | 0.7523 | 0.9422 | 0.5252 | 0.9363 | – |
|  |  |  |  |  |  |  |  |  |  |  |  |  |
| **Figure 7C (Day 1)** | |  |  |  |  |  | **Figure 7C (Day 21)** | |  |  |  |  |
| Group | Significance | Average |  |  |  |  | Group | Significance | Average |  |  |  |
| SD | A | 13.823333 |  |  |  |  | SD | A | 12.935 |  |  |  |
| HFD | A | 12.786667 |  |  |  |  | HFD | A | 12.21 |  |  |  |
| HFD+EC | A | 13.276667 |  |  |  |  | HFD+EC | A | 14.288333 |  |  |  |
| HFD+PCA | A | 11.678333 |  |  |  |  | HFD+PCA | A | 14.861667 |  |  |  |
| HFD+C3G | A | 12.285 |  |  |  |  | HFD+C3G | A | 12.878333 |  |  |  |
|  |  |  |  |  |  |  |  |  |  |  |  |  |
| P value (vs.) | SD | HFD | HFD+EC | HFD+PCA | HFD+C3G |  | P value (vs.) | SD | HFD | HFD+EC | HFD+PCA | HFD+C3G |
| SD | – | – | – | – | – |  | SD | – | – | – | – | – |
| HFD | 0.7232 | – | – | – | – |  | HFD | 0.952 | – | – | – | – |
| HFD+EC | 0.9633 | 0.9753 | – | – | – |  | HFD+EC | 0.6774 | 0.2778 | – | – | – |
| HFD+PCA | 0.1038 | 0.6722 | 0.3301 | – | – |  | HFD+PCA | 0.3486 | 0.1008 | 0.9793 | – | – |
| HFD+C3G | 0.3668 | 0.973 | 0.754 | 0.9471 | – |  | HFD+C3G | 1 | 0.964 | 0.6438 | 0.321 | – |
|  |  |  |  |  |  |  |  |  |  |  |  |  |
| **Figure 7C (Day 3)** | |  |  |  |  |  | **Figure 7C (Day 28)** | |  |  |  |  |
| Group | Significance | Average |  |  |  |  | Group | Significance | Average |  |  |  |
| SD | A | 13.353333 |  |  |  |  | SD | A | 13.024 |  |  |  |
| HFD | A | 11.423333 |  |  |  |  | HFD | A | 14.601667 |  |  |  |
| HFD+EC | A | 11.245 |  |  |  |  | HFD+EC | A | 14.165 |  |  |  |
| HFD+PCA | A | 10.76 |  |  |  |  | HFD+PCA | A | 14.434 |  |  |  |
| HFD+C3G | A | 11.385 |  |  |  |  | HFD+C3G | A | 12.946667 |  |  |  |
|  |  |  |  |  |  |  |  |  |  |  |  |  |
| P value (vs.) | SD | HFD | HFD+EC | HFD+PCA | HFD+C3G |  | P value (vs.) | SD | HFD | HFD+EC | HFD+PCA | HFD+C3G |
| SD | – | – | – | – | – |  | SD | – | – | – | – | – |
| HFD | 0.4458 | – | – | – | – |  | HFD | 0.4263 | – | – | – | – |
| HFD+EC | 0.3592 | 0.9998 | – | – | – |  | HFD+EC | 0.7149 | 0.9858 | – | – | – |
| HFD+PCA | 0.1786 | 0.9757 | 0.9924 | – | – |  | HFD+PCA | 0.5754 | 0.9997 | 0.9981 | – | – |
| HFD+C3G | 0.4265 | 1 | 0.9999 | 0.9804 | – |  | HFD+C3G | 1 | 0.3339 | 0.6243 | 0.4838 | – |
|  |  |  |  |  |  |  |  |  |  |  |  |  |
| **Figure 7C (Day 7)** | |  |  |  |  |  |  |  |  |  |  |  |
| Group | Significance | Average |  |  |  |  |  |  |  |  |  |  |
| SD | A | 12.823333 |  |  |  |  |  |  |  |  |  |  |
| HFD | A | 12.175 |  |  |  |  |  |  |  |  |  |  |
| HFD+EC | A | 11.713333 |  |  |  |  |  |  |  |  |  |  |
| HFD+PCA | A | 11.42 |  |  |  |  |  |  |  |  |  |  |
| HFD+C3G | A | 11.695 |  |  |  |  |  |  |  |  |  |  |
|  |  |  |  |  |  |  |  |  |  |  |  |  |
| P value (vs.) | SD | HFD | HFD+EC | HFD+PCA | HFD+C3G |  |  |  |  |  |  |  |
| SD | – | – | – | – | – |  |  |  |  |  |  |  |
| HFD | 0.9755 | – | – | – | – |  |  |  |  |  |  |  |
| HFD+EC | 0.8492 | 0.9931 | – | – | – |  |  |  |  |  |  |  |
| HFD+PCA | 0.7086 | 0.9577 | 0.9988 | – | – |  |  |  |  |  |  |  |
| HFD+C3G | 0.8415 | 0.992 | 0.9991 | 1 | – |  |  |  |  |  |  |  |

| **Figure 7D** |  |  |  |  |  |  |  |  |  |  |  |  |
| --- | --- | --- | --- | --- | --- | --- | --- | --- | --- | --- | --- | --- |
|  |  |  |  |  |  |  |  |  |  |  |  |  |
| **Figure 7D (Day 0)** | |  |  |  |  |  | **Figure 7D (Day 14)** | |  |  |  |  |
| Group | Significance | Average |  |  |  |  | Group | Significance | Average |  |  |  |
| SD | A | 10.105 |  |  |  |  | SD | A | 10.845 |  |  |  |
| HFD | A | 10.371667 |  |  |  |  | HFD | B | 7.005 |  |  |  |
| HFD+EC | A | 10.223333 |  |  |  |  | HFD+EC | B | 7.681667 |  |  |  |
| HFD+PCA | A | 10.105 |  |  |  |  | HFD+PCA | B | 7.82 |  |  |  |
| HFD+C3G | A | 10.536667 |  |  |  |  | HFD+C3G | B | 7.04 |  |  |  |
|  |  |  |  |  |  |  |  |  |  |  |  |  |
| P value (vs.) | SD | HFD | HFD+EC | HFD+PCA | HFD+C3G |  | P value (vs.) | SD | HFD | HFD+EC | HFD+PCA | HFD+C3G |
| SD | – | – | – | – | – |  | SD | – | – | – | – | – |
| HFD | 0.9624 | – | – | – | – |  | HFD | 0.0037 | – | – | – | – |
| HFD+EC | 0.9982 | 0.9958 | – | – | – |  | HFD+EC | 0.0205 | 0.9515 | – | – | – |
| HFD+PCA | 1 | 0.9624 | 0.9982 | – | – |  | HFD+PCA | 0.0286 | 0.9091 | 0.9999 | – | – |
| HFD+C3G | 0.8181 | 0.9937 | 0.9342 | 0.8181 | – |  | HFD+C3G | 0.004 | 1 | 0.9598 | 0.9213 | – |
|  |  |  |  |  |  |  |  |  |  |  |  |  |
| **Figure 7D (Day 1)** | |  |  |  |  |  | **Figure 7D (Day 21)** | |  |  |  |  |
| Group | Significance | Average |  |  |  |  | Group | Significance | Average |  |  |  |
| SD | A | 11.166667 |  |  |  |  | SD | A | 10.586667 |  |  |  |
| HFD | B | 7.598333 |  |  |  |  | HFD | B | 6.795 |  |  |  |
| HFD+EC | AB | 9.696667 |  |  |  |  | HFD+EC | AB | 8.465 |  |  |  |
| HFD+PCA | B | 7.366667 |  |  |  |  | HFD+PCA | AB | 8.951667 |  |  |  |
| HFD+C3G | B | 7.836667 |  |  |  |  | HFD+C3G | AB | 8.481667 |  |  |  |
|  |  |  |  |  |  |  |  |  |  |  |  |  |
| P value (vs.) | SD | HFD | HFD+EC | HFD+PCA | HFD+C3G |  | P value (vs.) | SD | HFD | HFD+EC | HFD+PCA | HFD+C3G |
| SD | – | – | – | – | – |  | SD | – | – | – | – | – |
| HFD | 0.0056 | – | – | – | – |  | HFD | 0.001 | – | – | – | – |
| HFD+EC | 0.5728 | 0.2355 | – | – | – |  | HFD+EC | 0.1103 | 0.29 | – | – | – |
| HFD+PCA | 0.01 | 0.9993 | 0.1553 | – | – |  | HFD+PCA | 0.3095 | 0.1015 | 0.9759 | – | – |
| HFD+C3G | 0.0178 | 0.9992 | 0.3451 | 0.9886 | – |  | HFD+C3G | 0.1147 | 0.2809 | 1 | 0.9788 | – |
|  |  |  |  |  |  |  |  |  |  |  |  |  |
| **Figure 7D (Day 3)** | |  |  |  |  |  | **Figure 7D (Day 28)** | |  |  |  |  |
| Group | Significance | Average |  |  |  |  | Group | Significance | Average |  |  |  |
| SD | A | 10.6 |  |  |  |  | SD | A | 11.198 |  |  |  |
| HFD | B | 6.41 |  |  |  |  | HFD | B | 8.401667 |  |  |  |
| HFD+EC | B | 7.16 |  |  |  |  | HFD+EC | AB | 9.298333 |  |  |  |
| HFD+PCA | B | 6.935 |  |  |  |  | HFD+PCA | AB | 9.552 |  |  |  |
| HFD+C3G | B | 7.178333 |  |  |  |  | HFD+C3G | AB | 8.985 |  |  |  |
|  |  |  |  |  |  |  |  |  |  |  |  |  |
| P value (vs.) | SD | HFD | HFD+EC | HFD+PCA | HFD+C3G |  | P value (vs.) | SD | HFD | HFD+EC | HFD+PCA | HFD+C3G |
| SD | – | – | – | – | – |  | SD | – | – | – | – | – |
| HFD | 0.0032 | – | – | – | – |  | HFD | 0.0469 | – | – | – | – |
| HFD+EC | 0.0191 | 0.9466 | – | – | – |  | HFD+EC | 0.2844 | 0.8507 | – | – | – |
| HFD+PCA | 0.0114 | 0.9852 | 0.9994 | – | – |  | HFD+PCA | 0.4633 | 0.7353 | 0.9987 | – | – |
| HFD+C3G | 0.0199 | 0.942 | 1 | 0.9992 | – |  | HFD+C3G | 0.1615 | 0.9643 | 0.9965 | 0.9728 | – |
|  |  |  |  |  |  |  |  |  |  |  |  |  |
| **Figure 7D (Day 7)** | |  |  |  |  |  |  |  |  |  |  |  |
| Group | Significance | Average |  |  |  |  |  |  |  |  |  |  |
| SD | A | 10.768333 |  |  |  |  |  |  |  |  |  |  |
| HFD | B | 7.476667 |  |  |  |  |  |  |  |  |  |  |
| HFD+EC | B | 6.361667 |  |  |  |  |  |  |  |  |  |  |
| HFD+PCA | B | 6.485 |  |  |  |  |  |  |  |  |  |  |
| HFD+C3G | B | 7.281667 |  |  |  |  |  |  |  |  |  |  |
|  |  |  |  |  |  |  |  |  |  |  |  |  |
| P value (vs.) | SD | HFD | HFD+EC | HFD+PCA | HFD+C3G |  |  |  |  |  |  |  |
| SD | – | – | – | – | – |  |  |  |  |  |  |  |
| HFD | 0.015 | – | – | – | – |  |  |  |  |  |  |  |
| HFD+EC | 0.0008 | 0.7655 | – | – | – |  |  |  |  |  |  |  |
| HFD+PCA | 0.0011 | 0.8323 | 0.9999 | – | – |  |  |  |  |  |  |  |
| HFD+C3G | 0.0092 | 0.9996 | 0.8664 | 0.9158 | – |  |  |  |  |  |  |  |

| **Figure 7E** |  |  |  |  |  |  |  |  |  |  |  |  |
| --- | --- | --- | --- | --- | --- | --- | --- | --- | --- | --- | --- | --- |
|  |  |  |  |  |  |  |  |  |  |  |  |  |
| **Figure 7E (Day 0)** | |  |  |  |  |  | **Figure 7E (Day 14)** | |  |  |  |  |
| Group | Significance | Average |  |  |  |  | Group | Significance | Average |  |  |  |
| SD | A | 2.7833333 |  |  |  |  | SD | A | 2.18 |  |  |  |
| HFD | A | 3.1916667 |  |  |  |  | HFD | B | 5.5366667 |  |  |  |
| HFD+EC | A | 2.6266667 |  |  |  |  | HFD+EC | B | 5.7516667 |  |  |  |
| HFD+PCA | A | 2.695 |  |  |  |  | HFD+PCA | B | 4.745 |  |  |  |
| HFD+C3G | A | 3.055 |  |  |  |  | HFD+C3G | B | 4.6916667 |  |  |  |
|  |  |  |  |  |  |  |  |  |  |  |  |  |
| P value (vs.) | SD | HFD | HFD+EC | HFD+PCA | HFD+C3G |  | P value (vs.) | SD | HFD | HFD+EC | HFD+PCA | HFD+C3G |
| SD | – | – | – | – | – |  | SD | – | – | – | – | – |
| HFD | 0.6826 | – | – | – | – |  | HFD | <.0001 | – | – | – | – |
| HFD+EC | 0.986 | 0.3826 | – | – | – |  | HFD+EC | <.0001 | 0.9961 | – | – | – |
| HFD+PCA | 0.9984 | 0.5088 | 0.9994 | – | – |  | HFD+PCA | 0.0019 | 0.6766 | 0.4582 | – | – |
| HFD+C3G | 0.9026 | 0.9916 | 0.6436 | 0.772 | – |  | HFD+C3G | 0.0024 | 0.6223 | 0.4074 | 1 | – |
|  |  |  |  |  |  |  |  |  |  |  |  |  |
| **Figure 7E (Day 1)** | |  |  |  |  |  | **Figure 7E (Day 21)** | |  |  |  |  |
| Group | Significance | Average |  |  |  |  | Group | Significance | Average |  |  |  |
| SD | A | 2.6566667 |  |  |  |  | SD | A | 2.3483333 |  |  |  |
| HFD | B | 5.1883333 |  |  |  |  | HFD | B | 5.415 |  |  |  |
| HFD+EC | AB | 3.58 |  |  |  |  | HFD+EC | B | 5.8233333 |  |  |  |
| HFD+PCA | AB | 4.3116667 |  |  |  |  | HFD+PCA | B | 5.91 |  |  |  |
| HFD+C3G | B | 4.4483333 |  |  |  |  | HFD+C3G | AB | 4.3966667 |  |  |  |
|  |  |  |  |  |  |  |  |  |  |  |  |  |
| P value (vs.) | SD | HFD | HFD+EC | HFD+PCA | HFD+C3G |  | P value (vs.) | SD | HFD | HFD+EC | HFD+PCA | HFD+C3G |
| SD | – | – | – | – | – |  | SD | – | – | – | – | – |
| HFD | 0.0022 | – | – | – | – |  | HFD | 0.004 | – | – | – | – |
| HFD+EC | 0.5407 | 0.0821 | – | – | – |  | HFD+EC | 0.0011 | 0.9829 | – | – | – |
| HFD+PCA | 0.0698 | 0.5887 | 0.7349 | – | – |  | HFD+PCA | 0.0008 | 0.9656 | 1 | – | – |
| HFD+C3G | 0.0426 | 0.7269 | 0.5973 | 0.9993 | – |  | HFD+C3G | 0.0861 | 0.6748 | 0.3609 | 0.3048 | – |
|  |  |  |  |  |  |  |  |  |  |  |  |  |
| **Figure 7E (Day 3)** | |  |  |  |  |  | **Figure 7E (Day 28)** | |  |  |  |  |
| Group | Significance | Average |  |  |  |  | Group | Significance | Average |  |  |  |
| SD | A | 2.7533333 |  |  |  |  | SD | A | 1.826 |  |  |  |
| HFD | B | 5.0133333 |  |  |  |  | HFD | C | 6.2 |  |  |  |
| HFD+EC | AB | 4.085 |  |  |  |  | HFD+EC | BC | 4.8666667 |  |  |  |
| HFD+PCA | AB | 3.825 |  |  |  |  | HFD+PCA | BC | 4.882 |  |  |  |
| HFD+C3G | AB | 4.2066667 |  |  |  |  | HFD+C3G | B | 3.9616667 |  |  |  |
|  |  |  |  |  |  |  |  |  |  |  |  |  |
| P value (vs.) | SD | HFD | HFD+EC | HFD+PCA | HFD+C3G |  | P value (vs.) | SD | HFD | HFD+EC | HFD+PCA | HFD+C3G |
| SD | – | – | – | – | – |  | SD | – | – | – | – | – |
| HFD | 0.031 | – | – | – | – |  | HFD | <.0001 | – | – | – | – |
| HFD+EC | 0.365 | 0.6966 | – | – | – |  | HFD+EC | <.0001 | 0.0858 | – | – | – |
| HFD+PCA | 0.5751 | 0.4767 | 0.9961 | – | – |  | HFD+PCA | <.0001 | 0.1162 | 1 | – | – |
| HFD+C3G | 0.2826 | 0.7918 | 0.9998 | 0.9831 | – |  | HFD+C3G | 0.0035 | 0.0013 | 0.3819 | 0.412 | – |
|  |  |  |  |  |  |  |  |  |  |  |  |  |
| **Figure 7E (Day 7)** | |  |  |  |  |  |  |  |  |  |  |  |
| Group | Significance | Average |  |  |  |  |  |  |  |  |  |  |
| SD | A | 2.055 |  |  |  |  |  |  |  |  |  |  |
| HFD | B | 4.6983333 |  |  |  |  |  |  |  |  |  |  |
| HFD+EC | B | 5.3516667 |  |  |  |  |  |  |  |  |  |  |
| HFD+PCA | B | 4.935 |  |  |  |  |  |  |  |  |  |  |
| HFD+C3G | B | 4.4133333 |  |  |  |  |  |  |  |  |  |  |
|  |  |  |  |  |  |  |  |  |  |  |  |  |
| P value (vs.) | SD | HFD | HFD+EC | HFD+PCA | HFD+C3G |  |  |  |  |  |  |  |
| SD | – | – | – | – | – |  |  |  |  |  |  |  |
| HFD | 0.001 | – | – | – | – |  |  |  |  |  |  |  |
| HFD+EC | <.0001 | 0.7902 | – | – | – |  |  |  |  |  |  |  |
| HFD+PCA | 0.0004 | 0.9938 | 0.95 | – | – |  |  |  |  |  |  |  |
| HFD+C3G | 0.0034 | 0.9874 | 0.4988 | 0.8938 | – |  |  |  |  |  |  |  |

**
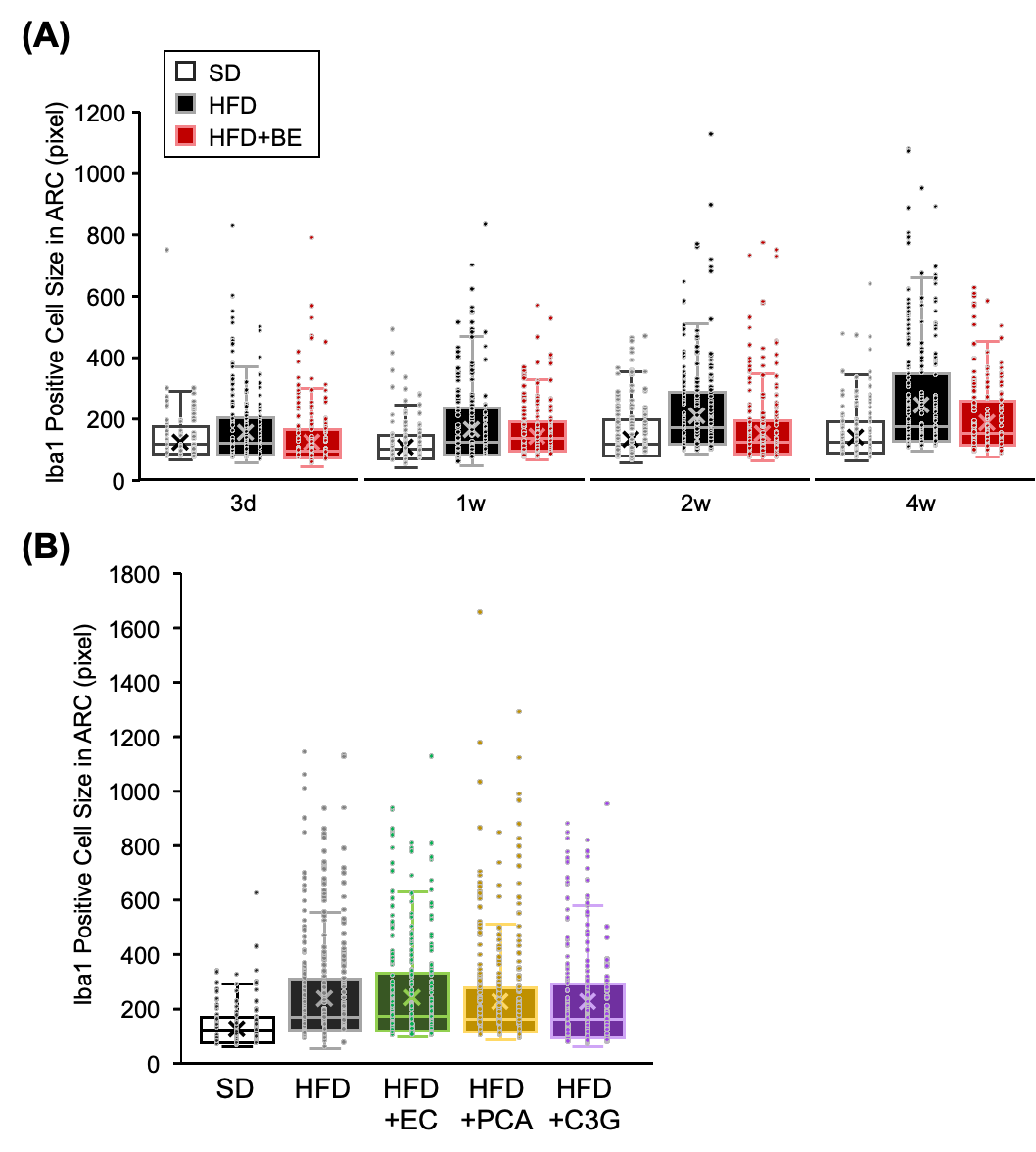
**

**Supplementary Figure 1. Dot plotting chart and box-and-whisker plots showing the size distribution of microglia in the ARC.** The dots mean microglial area distribution in each mouse. The box denotes the middle 50 percentile with mean (×) and median (–). Error bars denote maximum and minimum of distribution. **(A)** related to Figure 1A-C and **(B)** related to Figure 6A-C.

**
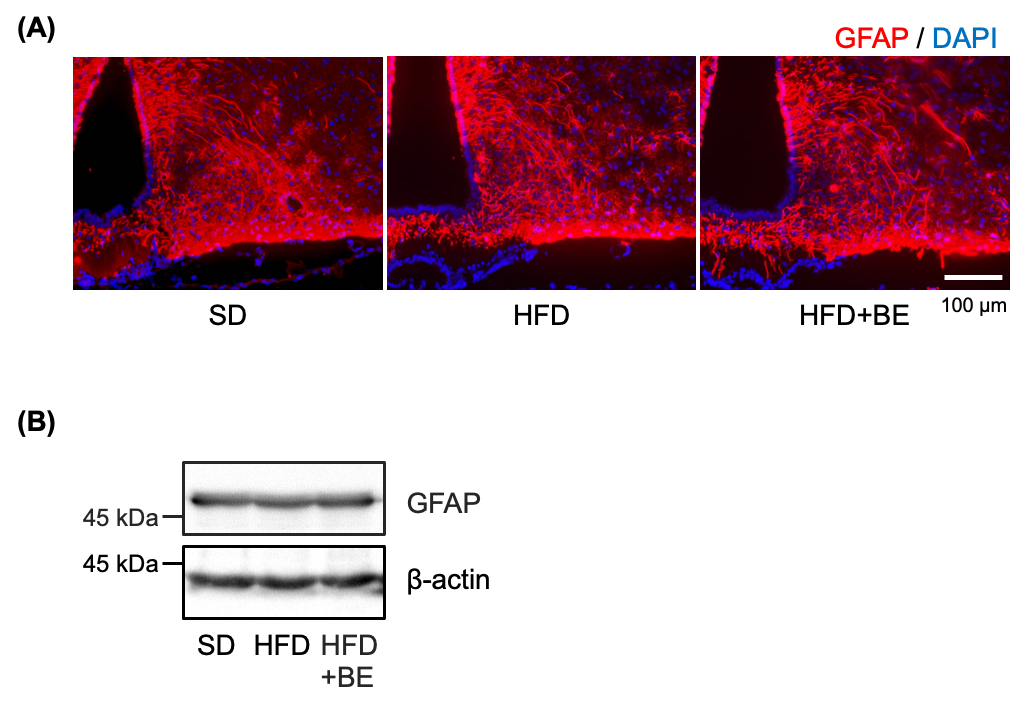
**

**Supplementary Figure 2. GFAP expression in the hypothalamus in mice of week 4 of the study (*Experiment 1*). (A)** Sections of the mediobasal hypothalamus of mice (10-µm-thick) were immunofluorescence-stained for glial fibrillary acidic protein (GFAP) with anti-GFAP antibody (*red*) and nuclei with 4',6-diamidino-2-phenylindole (DAPI) (*blue*) 4 weeks after the feedings. The left side hypothalamic arcuate nucleus (ARC) in each group are shown. **(B)** Western blotting analysis of GFAP and β-actin expression in the hypothalamus of mice 4 weeks after the feedings.

**
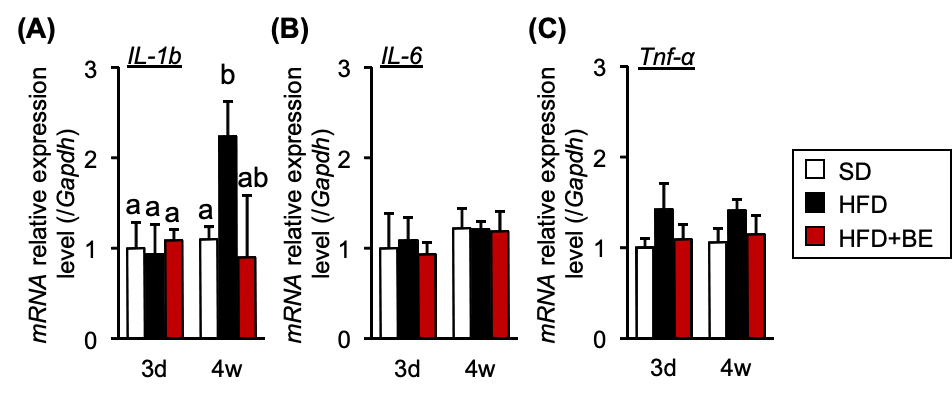
**

**Supplementary Figure 3.** **mRNA expression of pro-inflammatory cytokine genes in the epididymal adipose tissue of mice in *Experiment 1*.** mRNA expression of pro-inflammatory cytokine genes, **(A)** *Il-1b*, **(B)** *Il-6*, and **(C)** *Tnf-α*. Data shown are mean ± SE (*n* = 5). Different letters represent significant differences (*p* < 0.05) by Tukey-Kramer honestly significant difference test.


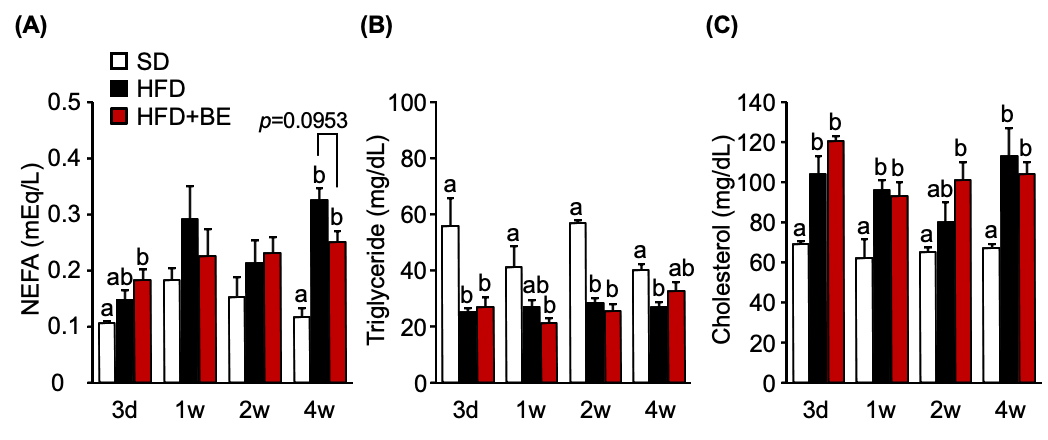


**Supplementary Figure 4. Plasma lipid levels in *Experiment 1*.** The concentration of **(A)** non-esterified fatty acid (NEFA), **(B)** triglyceride, and **(C)** total cholesterol in the plasma of mice on day 3, week 1, week 2, and week 4 of the feedings. Data shown are mean ± SE (*n* = 4–5). Different letters represent significant differences (*p* < 0.05) by Tukey-Kramer honestly significant difference test.


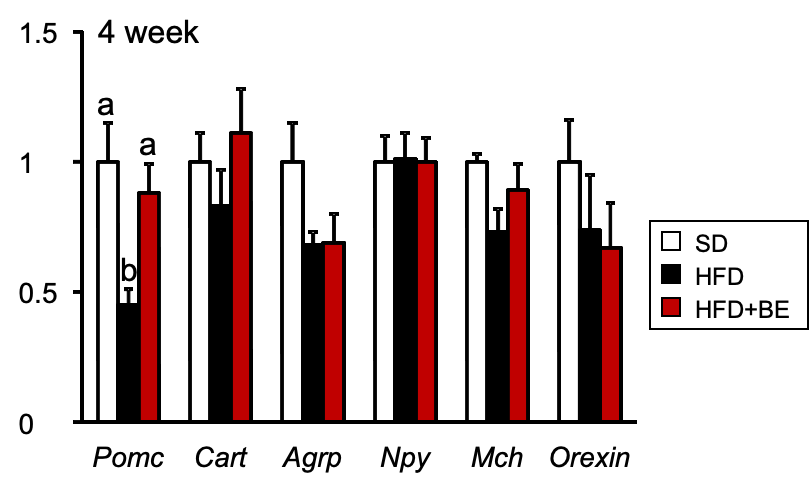


**Supplementary Figure 5. mRNA expression of hypothalamic neuropeptides regulating orexigenic and anorexigenic factors (*Experiment 1*).** mRNA expression of genes encoding hypothalamic neuropeptides regulating orexigenic and anorexigenic factors [*pro-opiomelanocortin* (*Pomc*), *cocaine- and amphetamine-regulated transcript* (*Cart*), *agouti-related protein* (*Agrp*), *neuropeputide Y* (*Npy*), *melanin-concentrating hormone* (*Mch*), and *orexin*] 4 weeks after the feedings. Data shown are mean ± SE (*n* = 5). Different letters represent significant differences (*p* < 0.05) by Tukey-Kramer honestly significant difference test.


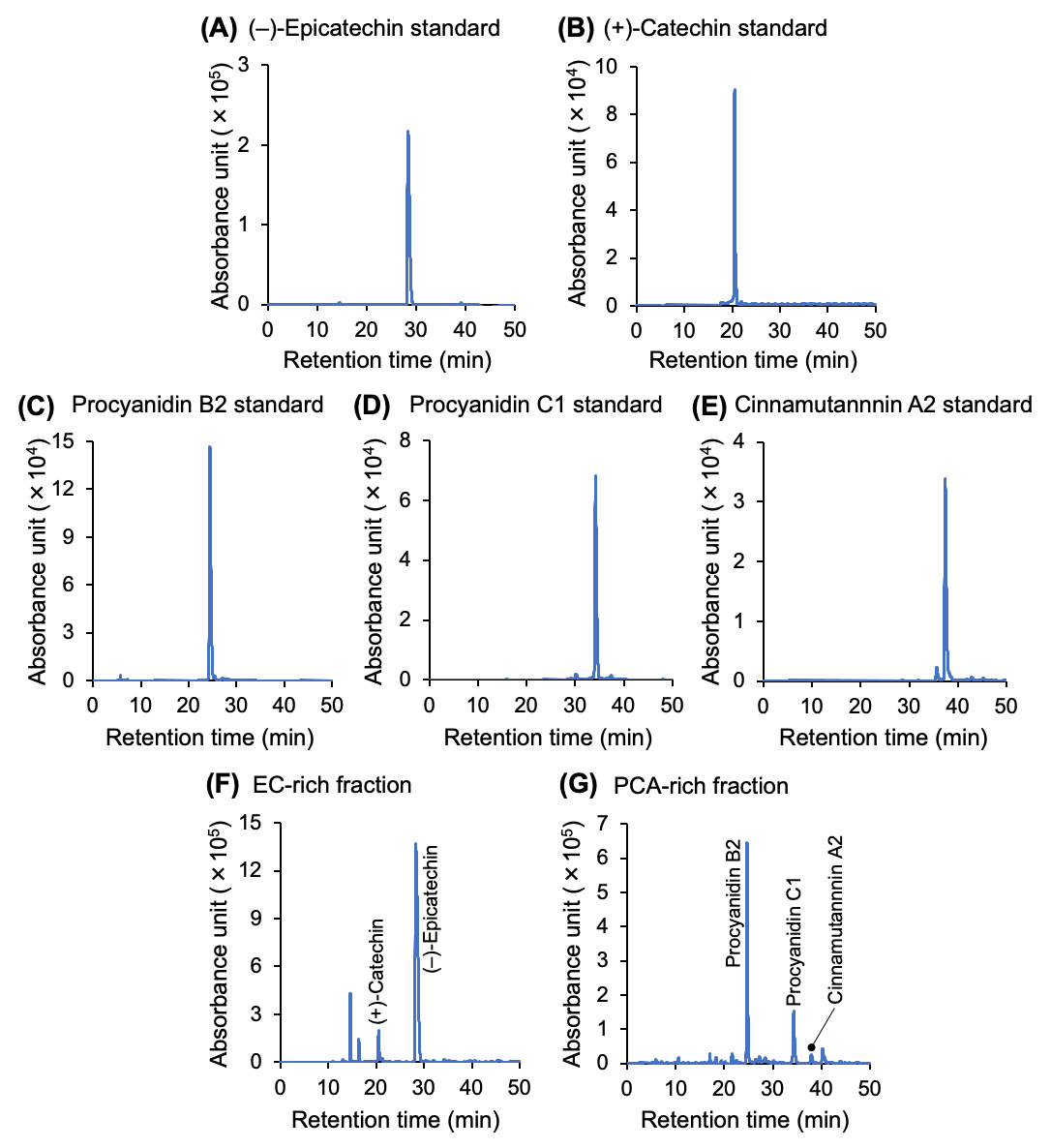


**Supplementary Figure 6. Typical HPLC chromatogram of (A-E) flavan 3-ols standards and (F and G) prepared fractions. (A)** (–)-epicatechin, **(B)** (+)-catechin, **(C)** procyanidin B2, **(D)** procyanidin C1, **(E)** cinnamutannnin A2, **(F)** EC-rich fraction, and **(G)** PCA-rich fraction. These compounds and fractions were analyzed by HPLC with a detection wavelength at 280 nm as described in the Section 2.2.

**
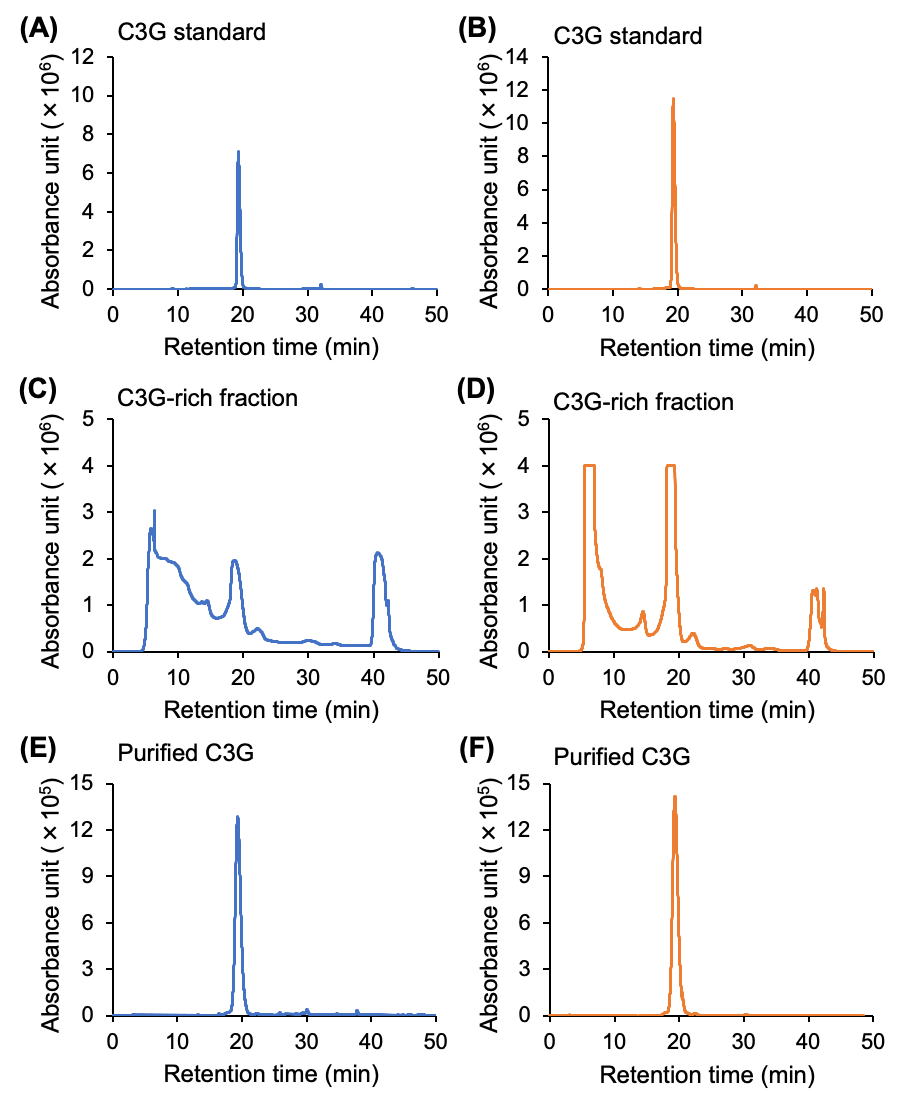
**

**Supplementary Figure 7. Typical HPLC chromatogram of (A and B) C3G standard, (C and D) aqueous fraction from ethyl acetate extraction, (E and F), and obtained C3G.** C3G and fraction were analyzed by HPLC with a detection wavelength at (A, C, and E) 280 nm (blue lines) and (B, D, and F) 513 nm (orange lines) as described in the Section 2.3.
